# ER-export and ARFRP1/AP-1–dependent delivery of SARS-CoV-2 Envelope to lysosomes controls late stages of viral replication

**DOI:** 10.1126/sciadv.adl5012

**Published:** 2024-04-03

**Authors:** Guy J. Pearson, Harriet V. Mears, Malgorzata Broncel, Ambrosius P. Snijders, David L. V. Bauer, Jeremy G. Carlton

**Affiliations:** ^1^ Organelle Dynamics Laboratory, The Francis Crick Institute, 1 Midland Road, London NW1 1AT, UK.; ^2^ School of Cancer & Pharmaceutical Sciences, King’s College London, Great Maze Pond, London SE1 1UL, UK.; ^3^ RNA Virus Replication Laboratory, The Francis Crick Institute, 1 Midland Road, London NW1 1AT, UK.; ^4^ Proteomic Science Technology Platform, The Francis Crick Institute, 1 Midland Road, London NW1 1AT, UK.

## Abstract

The β-coronavirus severe acute respiratory syndrome coronavirus 2 (SARS-CoV-2) is the causative agent of the global COVID-19 pandemic. Coronaviral Envelope (E) proteins are pentameric viroporins that play essential roles in assembly, release, and pathogenesis. We developed a nondisruptive tagging strategy for SARS-CoV-2 E and find that, at steady state, it localizes to the Golgi and to lysosomes. We identify sequences in E, conserved across *Coronaviridae*, responsible for endoplasmic reticulum–to–Golgi export, and relate this activity to interaction with COP-II via SEC24. Using proximity biotinylation, we identify an ADP ribosylation factor 1/adaptor protein–1 (ARFRP1/AP-1)–dependent pathway allowing Golgi-to-lysosome trafficking of E. We identify sequences in E that bind AP-1, are conserved across β-coronaviruses, and allow E to be trafficked from Golgi to lysosomes. We show that E acts to deacidify lysosomes and, by developing a trans-complementation assay for SARS-CoV-2 structural proteins, that lysosomal delivery of E and its viroporin activity is necessary for efficient viral replication and release.

## Teaser

SARS-CoV-2 Envelope exploits host cell endomembrane trafficking pathways to deacidify lysosomes and enhance viral replication.

## INTRODUCTION

Severe acute respiratory syndrome coronavirus 2 (SARS-CoV-2) is an enveloped, β-coronavirus with a positive-sense RNA genome encoding at least 29 different proteins (*1*). Late events in the β-coronaviral life cycle are orchestrated by four of these proteins—the RNA binding protein nucleocapsid (N) and the three transmembrane proteins Spike (S), Membrane (M), and Envelope (E). Viral assembly occurs on internal membranes and involves the budding of nascent particles into the secretory pathway lumen (*2*, *3*). The structural proteins M and E are thought to be necessary for viral budding (*4*, *5*), with incorporation of N allowing packaging of the viral genome (*6*). At steady state, coronaviral E proteins are known to localize to Golgi membranes, sites of coronaviral particle assembly (*3*, *7*, *8*). E is predicted to form a pentameric cation channel (*9*, *10*) and is only a minor component of coronavirus virions (*11*), suggesting that it plays important roles in manipulating the biology of the host. The channel activity of E contributes to acute respiratory distress syndrome (ARDS)–like pathological damage of E-expressing cells in both cellular and animal models (*10*). Recombinant coronaviruses lacking E exhibit defects in viral maturation and replication. For example, a SARS-CoV that lacks the E gene is attenuated in vitro and in vivo (*12*), a recombinant murine hepatitis virus (MHV) lacking E can replicate but produces smaller plaques in vitro (*13*), and a recombinant transmissible gastroenteritis virus (TGEV) lacking E is blocked in viral release with virions retained in the secretory pathway (*14*). These data suggest that E controls late events in the coronavirus life cycle that allow virus production and maturation (*3*). While viral egress was assumed to occur via the canonical secretory pathway, recent data suggest that β-coronaviruses can be delivered to deacidified lysosomes for atypical secretion via lysosomal exocytosis (*15*). Expression of E has been shown to cause deacidification of lysosomes (*16*) and a mutation (E^T9I^) in currently circulating omicron (B.1.1.529) variants that eliminate a polar pore-lining residue, compromise lysosomal deacidification, and lead to a reduced viral load (*17*), which is suggested to contribute to the reduced pathogenicity of this variant. Here, we asked how E was delivered to lysosomes to exert these effects. We identify sequence elements conserved across β-coronaviral E proteins that allow engagement with transport machineries allowing both endoplasmic reticulum (ER)–to–Golgi traffic and Golgi-to-lysosome traffic, and we identify the Golgi-localized guanosine triphosphatase (GTPase) adenosine diphosphate ribosylation factor–related protein 1 (ARFRP1) as being essential for the recruitment of adaptor protein–1 (AP-1) to the Golgi and for coordinating an AP-1–dependent trafficking route for delivering E to lysosomes. By developing a trans-complementation assay for E subgenomic mRNA (sgmRNA), we demonstrate the importance of this trafficking pathway for late stages of the SARS-CoV-2 life cycle.

## RESULTS

### Internally tagged SARS-CoV-2 Envelope traffics to and deacidifies lysosomes

**Fig. 1.**
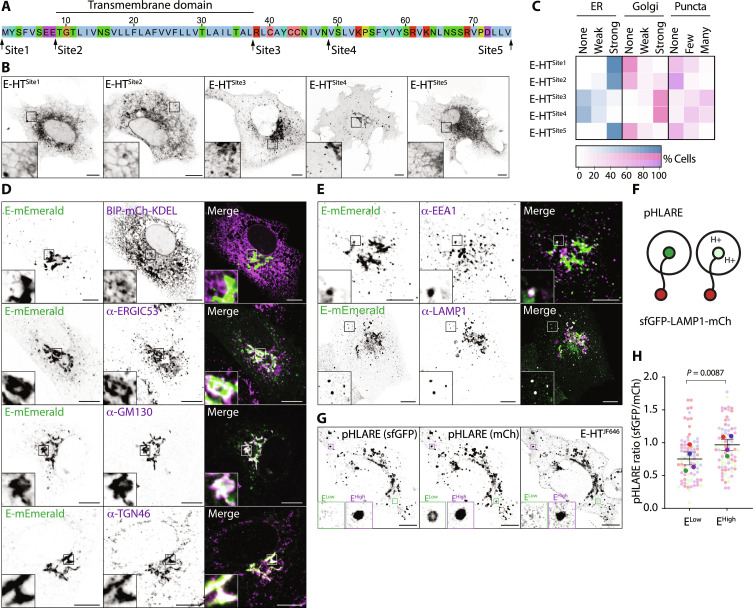
Placement of internal tags allows visualization of the trafficking itinerary of SARS-CoV-2 Envelope. (**A**) Sequence of Envelope (E) protein from SARS-CoV-2 indicating the position of internal insertion sites. Amino acids colored according to ClustalX criteria. (**B**) Representative live images of VeroE6 cells transfected with plasmids encoding the indicated Janelia Fluor 646 (JF646)–illuminated E-HT fusion proteins. (**C**) Quilt of the indicated HT-fusion proteins from 50 imaged cells in (B). (**D** and **E**) Representative images of VeroE6 cells transfected with plasmids encoding E-mEmerald, fixed and stained with antisera raised against ERGIC53, GM130, TGN46, EEA1, or LAMP1, or that were cotransfected with plasmids encoding BIP-mCh-KDEL. Arrowheads indicate colocalized E-mEmerald and LAMP1-tdTomato. Images representative of between 13 and 26 captured images in each case. (**F**) Schematic of pHLARE assay. (**G**) Representative image of VeroE6 cells expressing pHLARE and JF646-illuminated E-HT. Examples of E-HT–low and E-HT–high lysosomes are displayed. (**H**) Superplot of ratiometric imaging of sfGFP and mCherry in JF646-high and JF646-low lysosomes within the same cell. Each presented data point represents the mean sfGFP/mCh signal of each mCh-positive lysosome in the cell binned into high or low classes based on its JF646 signal. Means ± SE displayed from *n* = 1364 JF646-high and *n* = 2761 JF646-low pHLARE-positive structures from *N* = 28 cells across four independent experiments, with significance determined by paired two-tailed *t* test. In microscopy panels, scale bars are 10 μm.

As antisera capable of recognizing SARS-CoV-2 E are unavailable, we generated tagged versions of E to investigate its intracellular trafficking itinerary. We were surprised to find that N- or C-terminal HaloTag (HT) fusions restricted E to the ER (Fig. 1, A to C), the site of its biogenesis, suggesting that canonical tagging disrupts the proper localization of this protein. We found that placement of HT at internal positions either immediately after the transmembrane domain (E-HT^Site3^) or in a region of the cytoplasmic tail (E-HT^Site4^) of E allowed steady-state localization to Golgi membranes (Fig. 1, A to C), consistent with known localization for E proteins (*3*). We confirmed that localization of the fluorescent reporters was driven by E (fig. S1A) and found that mEmerald fusions localized similarly to HT (fig. S1B), and we devised a quantitative imaging–based localization table (QUILT) to depict E’s position within the secretory pathway. This quantification revealed broadly similar quantitative reports of E localization for tags placed at site 3 or site 4 (Fig. 1C and fig. S1C). Unless otherwise indicated, experiments hereafter use internal tags placed at Site3, with analysis performed after 16 to 18 hours of expression to limit the toxicity associated with expression of E (fig. S1D) (*10*).

We found good colocalization of E-mEmerald with 130-kDa cis-Golgi matrix protein (GM130) and the trans-Golgi Network (TGN) protein, TGN46 (Fig. 1D). We observed no colocalization with an mCherry targeted to the ER lumen, and we observed partial colocalization with the endogenous Golgi-localized pool of the ER-Golgi intermediate compartment (ERGIC) marker, ERGIC53. These data suggest that at steady state, E-mEmerald is exported efficiently from the ER and reaches the Golgi. In addition to the predominate perinuclear Golgi localization, we noticed that E-HT and E-mEmerald decorated punctate structures in the cytoplasm (Fig. 1, B to E). We found good colocalization of these peripheral puncta with lysosomal-associated membrane protein–1 (LAMP1), a marker of late endosomes and lysosomes, and occasional colocalization with early endosomal antigen 1 (EEA1) (Fig. 1E), suggesting that lysosomal access is gained via endosomes. We validated colocalization with endogenous Golgin97 and CD63 staining (fig. S1E), alternate markers of TGN and lysosomes. Both perinuclear and punctate localization of E-HT and E-mEmerald was confirmed in cells expressing all four SARS-CoV-2 structural proteins and in cells infected with SARS-CoV-2 (fig. S1, F and G), indicating that these localizations are preserved during particle assembly.

Coronaviral E proteins assemble into pentameric viroporins (*9*). We used fluorescence lifetime imaging–Forster radius energy transfer (FLIM-FRET) to confirm that the lifetime of E-mEmerald in post-Golgi vesicular structures was reduced in the presence of tetramethylrhodamine (TMR)–labeled E-HT, suggesting that E oligomerizes in these organelles (fig. S1, H and I). We transfected E-HT into VeroE6 cells and, using the recently described pH lysosomal activity reporter (pHLARE), which provides an internally controlled ratiometric report of the luminal environment sensed by LAMP1 (Fig. 1F and fig. S1J) (*18*), found that lysosomes containing higher levels of E-HT were deacidified relative to lysosomes containing lower levels of E-HT (Fig. 1, G and H). These data suggest that while E localizes predominantly to Golgi membranes, a pool of E is trafficked onward to lysosomes and allows pH neutralization in these organelles.

### A peptide motif in SARS-CoV-2 Envelope’s C terminus drives ER export

**Fig. 2.**
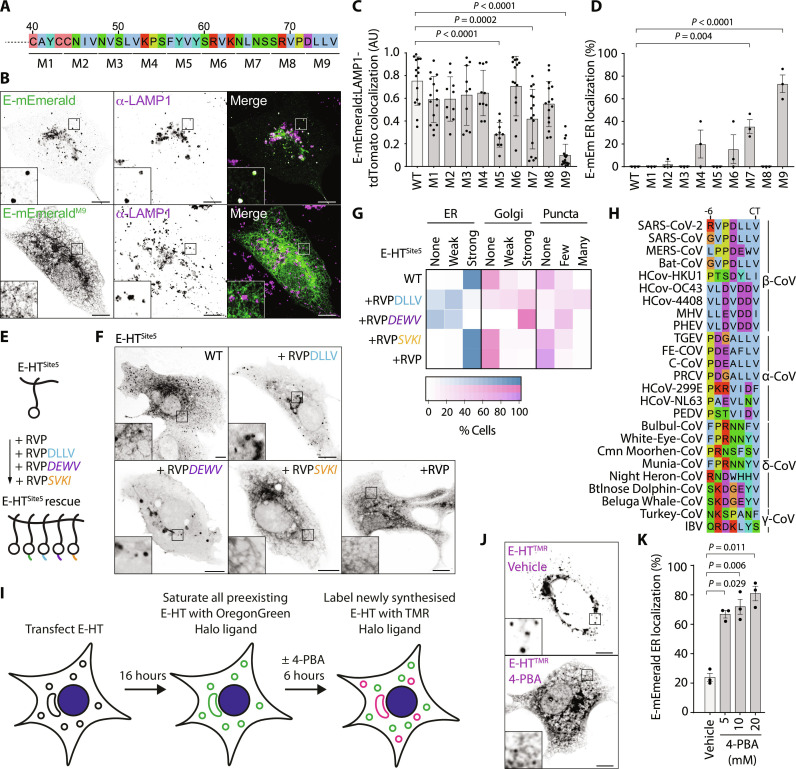
Alanine-scanning mutagenesis reveals C-terminal sequences necessary for ER-to-Golgi trafficking of E. (**A**) Schematic of alanine-scanning mutagenesis. In mutants M1 to M9, the indicated amino acids were exchanged for alanine. (**B**) Representative images of VeroE6 cells expressing the E-mEmerald or E-mEmerald^M9^ and stained against LAMP1. Images representative of 26 and 10 acquired cells. (**C** and **D**) Quantification of subcellular distribution of E-mEmerald M1 to M9. Overlap of E-mEmerald with LAMP1-tdTomato assessed by Manders’ correlation coefficient from all non-Golgi, E-positive regions of each cell. Fifteen imaged cells, with means ± SD displayed (C). ER localization (D) was scored visually from 15 imaged cells across three independent experiments, with means ± SE displayed. For (C) and (D), statistical significance determined by one-way analysis of variance (ANOVA) with Dunnett’s correction. (**E** and **F**) Cartoon of rescue assay (E) and representative images (F) of VeroE6 cells transfected with plasmids encoding the indicated JF646-illuminated E-HT^Site5^ fusions with C-terminal additions of the indicated chimeric terminal peptides. Plasmids transfected encoded E-HT^Site5^, E-HT^Site5^-RVPDLLV, E-HT^Site5^-RVP*DEWV* (MERS-CoV), E-HT^Site5^-RVP*SVKI* (class-II PDZ), or E-HT^Site5^-RVP. Images representative of between 9 and 19 imaged cells in each case. Chimeric sequences italicized, HT depicted as a circle. (**G**) Quilt displaying localization from 50 scored cells for each condition in (F). (**H**) Sequence alignment of the extreme C terminus of α, β, γ, and δ coronaviral E proteins. (**I**) Cartoon of E-HT pulse-chase assay. (**J**) Representative images of newly synthesized E-HT^R^ in the presence or absence of 10 mM 4-PBA for 6 hours. The full panel of images from this experiment is fig. S3G. (**K**) Quantification of cells displaying ER localization of newly synthesized E-HT^R^ from 50 imaged cells per experiment in three independent experiments imaged in (J). Means ± SE displayed, significance determined by one-way ANOVA with Dunnett’s correction. In microscopy panels, scale bars are 10 μm.

Transmembrane proteins are cotranslationally inserted into the ER. We next performed alanine-scanning mutagenesis through the cytosolic tail of E to identify sequences required for its trafficking to the Golgi and onward toward lysosomes in VeroE6 cells (Fig. 2A). We found that mutation of the C-terminal four amino acids to alanine (E-mEmerald^M9^) restricted E to its site of biosynthesis in the ER, prevented its localization to lysosomes (Fig. 2, A to D, and fig. S2, A to D), and confirmed ER retention of E-mEmerald^M9^ in A549, Caco-2, and Calu-3 cells (fig. S2E). Grafting these C-terminal amino acids onto E-HT^Site5^ restored anterograde traffic of this protein (Fig. 2, E to G), indicating that this sequence acts as a dominant ER-export motif and explains why C-terminal fusions of E are retained in the ER.

The C-terminal four amino acids of SARS-CoV E have also been described to encode a PSD95, Dlg1, ZO-1 (PDZ) ligand (*19*). This sequence is conserved in SARS-CoV-2 E, and we wondered whether engagement with a PDZ domain–containing partner licensed ER export of E. However, we found that sequences from a variety of different classes of PDZ ligands could substitute for the DLLV sequence and drive ER export, although none were as effective as chimeric C-termini from MHV (strain S) or Middle East respiratory syndrome (MERS)-CoV (fig. S3, A and B). The variety of ER export–competent PDZ ligands from different classes argues against a specific PDZ domain–containing protein being required for ER export. Consistent with models of Coat Protein Complex II (COP-II)–dependent ER export, the C-terminal valine of E provided most of the export activity, as E-mEmerald^ΔV^ was largely retained in the ER, and exchanging the terminal DLLV for AAAV (E-mEmerald^DLLV-AAAV^) restored ER export (fig. S3, C and D). However, a small pool of E-mEmerald^ΔV^ still reached the Golgi, suggesting that the context of this hydrophobic valine is important for ER export. C-terminal hydrophobic residues are a conserved feature of E proteins (Fig. 2H), suggesting that ER export may be used across *Coronaviridae* to access the Golgi for viral assembly. The beta variant (B.1.351) of SARS-CoV-2 encodes E^P71L^, and we wondered whether this mutation influenced the efficiency of ER export. While E-mEmerald^P71L^ displayed steady-state localization to the Golgi, a fraction was retained in the ER, and its ability to reach post-Golgi structures was limited (fig. S3, E and F), suggesting that impaired ER export may be a feature of some previously circulating variants of SARS-CoV-2. C-terminal hydrophobic ER-export signals in secretory cargo proteins are typically recognized by the B site of SEC24 isoforms (*20*) for incorporation into the COP-II coat. Using a pulse-chase assay with sequentially applied HT ligands (Fig. 2I), we found that 4-phenylbutyric acid (4-PBA), a small molecule that occludes the SEC24 B site (*21*), suppressed ER export of newly synthesized E-HT (Fig. 2, J and K, and fig. S3G).

### Proximity biotinylation identifies host factors interacting with SARS-CoV-2 Envelope

We next inserted a hemagglutinin (HA)–tagged TurboID (*22*) into the internal tagging sites in E and confirmed that this did not disrupt E’s localization (fig. S4A). After confirming that versions of E-HA/TurboID colocalized with E-mEmerald in 293T cells (fig. S4B), we used proximity biotinylation, mass spectrometry (MS), and label-free quantification (LFQ) to determine the proximal proteome of E in these cells (fig. S4, C to E, and data S1 to S3). We found that many ERGIC and Golgi proteins and components of both anterograde (SEC24B) and retrograde [retention in endoplasmic reticulum sorting receptor 1 (RER1), Coatomer subunit epsilon] transport machineries were significantly enriched by E-HA/TurboID, relative to a cytosolic control (fig. S4F). We confirmed physical interactions with RER1, Golgi reassembly stacking protein 2 (GRASP55), and PALS-1, a previously identified SARS-CoV E–and SARS-CoV-2 E–interacting partner (fig. S4F) (*23*, *24*). We next compared proximal proteomes from ER export–proficient (E-HA/TurboID^Site3^, E-HA/TurboID^Site4^, and E-HA/TurboID^Site3ΔDLLV+DEWV^) and ER export–defective (E-HA/TurboID^Site3ΔDLLV^, E-HA/TurboID^Site4ΔDLLV^, and E-HA/TurboID^Site3ΔDLLV+SVKI^) versions of E. Reported proximal proteomes from these differentially localized versions of E clustered well by principal components analysis and hierarchical clustering (figs. S4C and S5, A and B). We recovered peptides from numerous PDZ domain–containing proteins with wild-type (WT) but not ΔDLLV versions of E-HA/TurboID (fig. S5, C and D), confirming that this sequence can act as a PDZ ligand. We observed enrichment of Golgi and ERGIC proteins for ER export–competent versions of E-HA/TurboID and enrichment of ER proteins for versions of E lacking the ability to escape the ER (fig. S5D). Consistent with our identification of COP-II–dependent ER export (Fig. 2), we recovered the COP-II components SEC24A, SEC24B, and SEC31A with ER export–competent versions of E (fig. S5D). Last, in agreement with our imaging approaches documenting the localization of E to lysosomes, we detected significant enrichment of endosomal and lysosomal proteins in our ER export–competent versions of E (fig. S5D). When compared to previously published proteomes for E (*1*, *25*–*29*), our internally tagged versions of E report more candidates and a larger proportion of Golgi and endolysosomal proteins than N- or C-tagged versions (data S4). We identify here an extensive set of interaction partners for SARS-CoV-2 E across biosynthetic and endocytic pathways.

### ARFRP1 and AP-1 allow Golgi-to-lysosome trafficking of SARS-CoV-2 Envelope

**Fig. 3.**
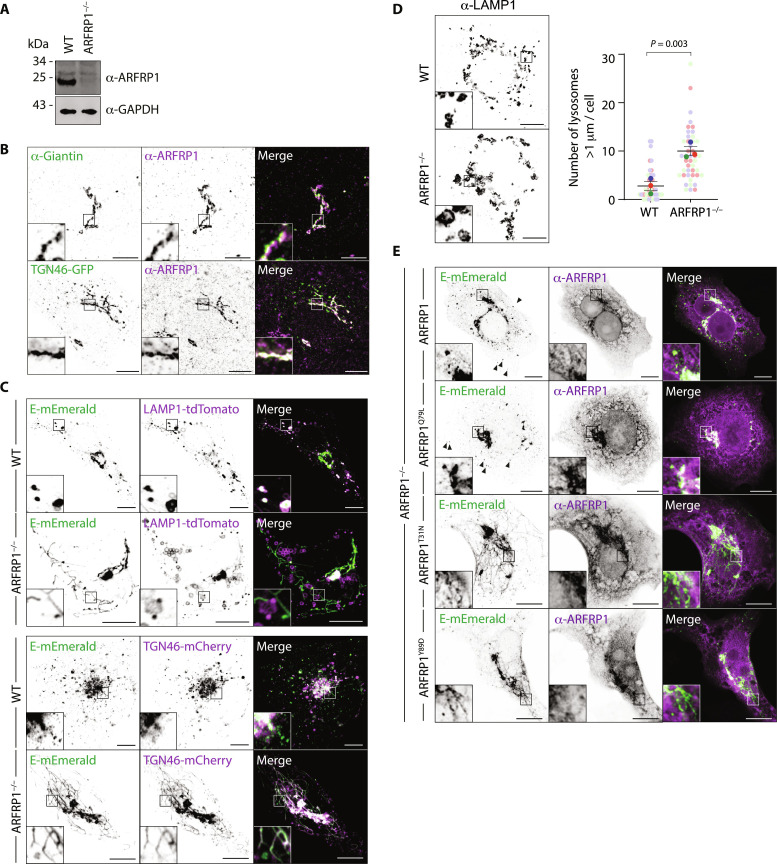
ARFRP1 controls Golgi-to-lysosome delivery of SARS-CoV-2 Envelope. (**A**) Resolved lysates from WT or ARFRP1^−/−^ VeroE6 cells were examined by Western blotting with antibodies raised against ARFRP1 or glyceraldehyde-3-phosphate dehydrogenase (GAPDH). (**B**) VeroE6 cells or VeroE6 cells transfected with a plasmid encoding TGN46-EGFP were fixed and stained with antisera raised against Giantin and/or ARFRP1. Images representative of 11 imaged cells in each case. (**C**) Representative images of WT or ARFRP1^−/−^ VeroE6 cells transfected with plasmids encoding E-mEmerald and either LAMP1-tdTomato or TGN46-mCherry. Images representative of between 11 and 19 imaged cells in each case. (**D**) Representative images and superplot quantification of lysosome size in WT or ARFRP1^−/−^ VeroE6 cells stained with antisera raised against LAMP1. The number of lysosomes >1 μm in diameter (*64*) was quantified from 45 imaged cells acquired across three independent experiments, with mean ± SE displayed and significance calculated by a paired two-tailed *t* test. (**E**) Representative images of ARFRP1^−/−^ VeroE6 cells transfected with plasmids encoding E-mEmerald and either ARFRP1, ARFRP1^Q79L^, ARFRP1^T31N^, or ARFRP1^Y89D^. Cells were stained with antibodies raised against ARFRP1 to detect transfected cells. Images representative of five imaged cells in each case. Arrowheads display localization of E-mEmerald to peripheral puncta. In microscopy panels, scale bars are 10 μm.

**Fig. 4.**
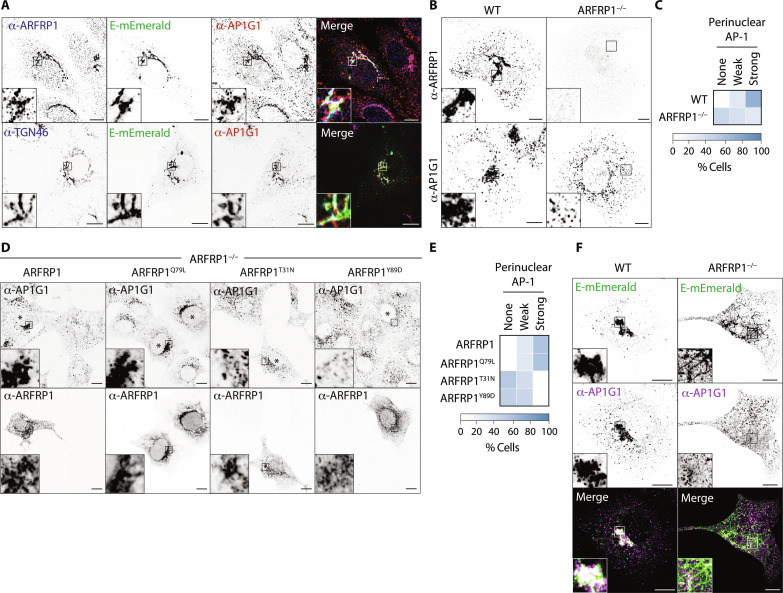
ARFRP1 controls AP-1 localization to Golgi membranes and Golgi-to-lysosome export of SARS-CoV-2 Envelope. (**A**) VeroE6 cells were transfected with a plasmid encoding E-mEmerald and stained with antibodies raised against endogenous AP1G1 and ARFRP1, or endogenous AP1G1 and TGN46. Images representative of 25 and 15 imaged cells, respectively. (**B** and **C**) WT or ARFRP1^−/−^ VeroE6 cells were fixed and stained with antisera raised against ARFRP1 or AP1G1, and perinuclear localization of AP1G1 was scored in the accompanying quilt from 50 imaged cells (C). (**D** and **E**) Plasmids encoding the indicated ARFRP1 proteins were transfected into ARFRP1^−/−^ VeroE6 cells. Cells were fixed and stained with antisera raised against ARFRP1 or AP1G1, and the perinuclear localization of AP1G1 was scored in the accompanying quilt from over 50 imaged cells per condition across three independent experiments (E). Transfected cells indicated by asterisks. (**F**) WT or ARFRP1^−/−^ VeroE6 cells were transfected with a plasmid encoding E-mEmerald and stained with antibodies raised against endogenous AP1G1. Images representative of 14 or 23 imaged cells, respectively. In microscopy panels, scale bars are 10 μm.

We next questioned how E was delivered to lysosomes. Some lysosomal proteins are first delivered to the cell surface and then internalized via endocytic routes to allow lysosomal localization. Alternatively, the heterotetrameric clathrin adaptor complex, AP-1, can select cargo for TGN-to-endosome transport, where it works in concert with the Golgi-localized Gamma ear–containing adaptor–1 (GGA1) and AP1AR/Gadkin, a kinesin adaptor responsible for the anterograde movement of AP-1 carriers (*30*–*32*). An AP-3–dependent pathway is also thought to deliver cargo directly from Golgi to lysosomes, although this is less well characterized in mammalian cells (*33*). Expression of a dominant negative form of the endocytic GTPase, Dynamin (*34*), robustly blocked transferrin internalization but had no impact on the intracellular distribution of E-mEmerald (fig. S6, A and B), suggesting that E is not internalized from the plasma membrane. To investigate host-cell factors responsible for Golgi export of E, we selected 12 membrane trafficking genes identified as high-confidence hits from our proximal proteome (fig. S6C) and used CRISPR-Cas9 to delete them in VeroE6 cells. Most of these candidates were similarly enriched if we compared ER export–proficient to ER export–defective versions of E (fig. S6D), and we observed strong correlation of hits identified with labeling at site 3 or site 4 (fig. S6E). We verified homozygous deletion for each target by next-generation sequencing or Western blotting (Fig. 3A and fig. S7, A and B) and compared localization of E-mEmerald in these lines (fig. S7, C and D). E-mEmerald localized to ARFRP1-positive membranes at the Golgi (fig. S7E). ARFRP1 is a TGN-resident ARF1-related GTPase (*35*), and we found that endogenous ARFRP1 colocalized with TGN46–green fluorescent protein (GFP) and that ARFRP1-positive membranes were juxtaposed against membranes positive for the cis- and medial-cisternae localizing golgin, Giantin (Fig. 3B). In ARFRP1^−/−^ cells, we found that while E-mEmerald was able to exit the ER, it was retained in TGN46-mCherry–positive tubules emanating from the Golgi and did not reach the lysosomes (Fig. 3C and fig. S7, C and D). We illuminated endogenous LAMP1 and confirmed that E-mEmerald no longer localized to lysosomes in ARFRP1^−/−^ VeroE6 cells (fig. S8, A and B). E-mEmerald was instead retained in tubular structures that were decorated with endogenous GM130 and GRASP55 (fig. S8A). Although LAMP1-positive structures were swollen in ARFRP1^−/−^ cells (Fig. 3D), transmembrane proteins such as LAMP1 were correctly localized (Fig. 3C and fig. S8A), suggesting that these cells do not exhibit a global block in Golgi export. We next reexpressed versions of ARFRP1 in ARFRP1^−/−^ VeroE6 cells to test the requirements for its enzymatic activity in the Golgi export of E-mEmerald. Reexpression of ARFRP1 or its catalytically active mutant, ARFRP1^Q79L^, in ARFRP1^−/−^ cells restored export of E-mEmerald to peripheral puncta and suppressed its retention in Golgi-derived tubules. Reexpression of a dominant-negative mutant, ARFRP1^T31N^, or ARFRP1^Y89D^, a version of ARFRP1 containing a mutation in its hydrophobic effector patch equivalent to ARF1^Y81D^ (fig. S8C) (*36*), could not (Fig. 3E). Reexpression of ARFRP1, ARFRP1^T31N^, or ARFRP1^Q79L^ matched previously reported localizations of ARFRP1 (*35*), and localization of these proteins was not influenced by coexpression of E-mEmerald (fig. S8D). ARFRP1^Y89D^ and ARFRP1^T31N^ still localized to the Golgi but were not themselves incorporated into the E-mEmerald–containing Golgi-derived tubules (Fig. 3E), suggesting that ARFRP1 coordinates a machinery allowing carrier formation for Golgi-to-endosome trafficking of E. We interrogated our E-HA/TurboID proximal interactome and noted enrichment of members of the AP-1 clathrin adaptor complex, AP1AR/Gadkin and GGA1 (fig. S9A). ARFRP1 has been previously shown to interact with AP-1 in a guanosine triphosphate (GTP)–dependent manner and play roles in the TGN export of the planar cell polarity protein, Vangl2 (*37*). AP-1 has been identified as necessary for SARS-CoV-2 replication in several genome-wide CRISPR screens (*38*–*40*), but the mechanistic basis for its contribution to the SARS-CoV-2 life cycle remains unexplored. Endogenous AP-1 could be detected on ARFRP1-positive E-mEmerald–positive TGN membranes (Fig. 4A and fig. S9B). Loss of AP-1 via small interfering RNA (siRNA)–mediated depletion of AP1M1 phenocopied ARFRP1 deletion, with E-mEmerald retained in Golgi-derived tubules (fig. S9, C to E), suggesting that AP-1 and ARFRP1 operate in the same pathway to allow Golgi export of E. Depletion of AP1AR/Gadkin suppressed formation of these tubules, in both WT and ARFRP1^−/−^ VeroE6 cells (fig. S9, C to E), suggesting that they are generated via coupling to anterograde microtubule motors. Last, GGA1-depletion mimicked the loss of AP1M1 and similarly led to the retention of E-mEmerald in Golgi-derived tubular structures (fig. S9, C to E), suggesting that it operates alongside ARFRP1 and AP-1 in Golgi export of E-mEmerald. In the case of depletion of AP1M1 or GGA1 in ARFRP1^−/−^ cells, we observed no additive phenotypes in tubule formation (fig. S9E), suggesting that these proteins operate in the same pathway. We confirmed the retention of E-mEmerald in Golgi-derived tubules and the impaired lysosomal delivery in AP1M1-depleted A549 cells (fig. S9F). Given the similarities in E-mEmerald phenotypes produced upon the inactivation of ARFRP1 and AP-1, we next examined AP-1 localization in WT and ARFRP1^−/−^ cells. While AP-1 levels were identical in both cell lines (fig. S10A), AP-1 was delocalized from the perinuclear region in ARFRP1^−/−^ cells (Fig. 4, B and C). AP-1’s perinuclear localization could be restored in ARFRP1^−/−^ cells by reexpression of ARFRP1 or ARFRP1^Q79L^ but not by reexpression of ARFRP1^T31N^ or ARFRP1^Y89D^ (Fig. 4, D and E). In ARFRP1^−/−^ cells expressing E-mEmerald, AP-1 no longer localized to E-mEmerald–positive membranes at the Golgi (Fig. 4F). Last, we found that overexpression of ARFRP1^T31N^ or ARFRP1^Y89D^ could delocalize endogenous AP-1, suggesting that these mutants act as dominant-negative inhibitors of AP-1 at this organelle (fig. S10, B and C). These data identify ARFRP1 as a TGN-localized GTPase, whose activity is necessary for localizing AP-1 to this organelle, and reveal that an ARFRP1/AP-1–dependent pathway allows export of E from the Golgi and its delivery to lysosomes.

### SARS-CoV-2 Envelope binds AP-1

**Fig. 5.**
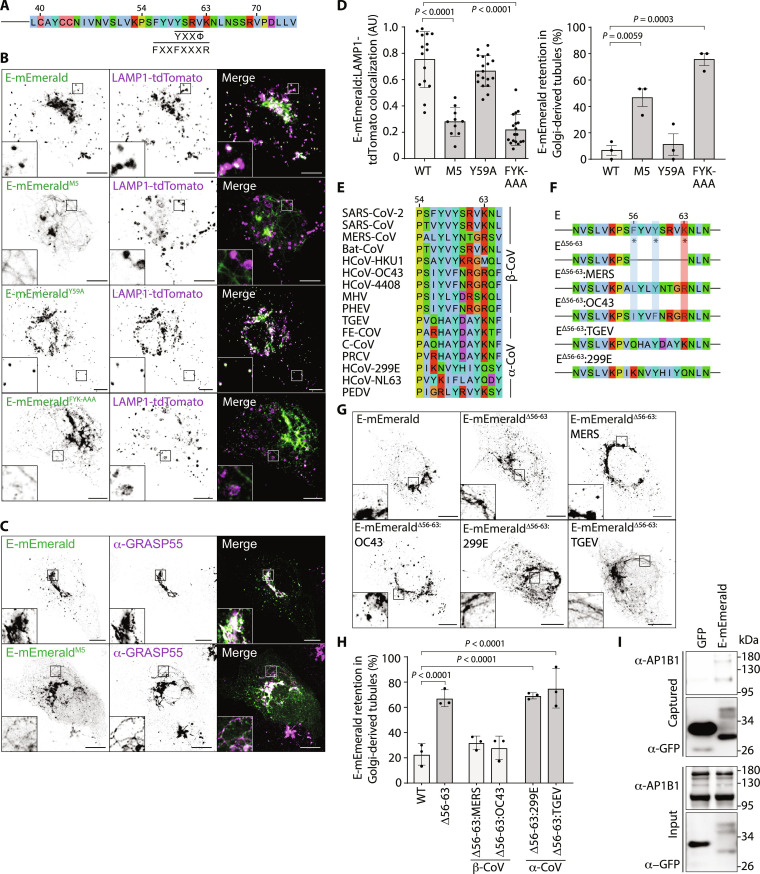
SARS-CoV-2 Envelope binds the AP-1 adaptor protein complex. (**A**) Schematic of E’s C terminus, with putative AP-binding SLIMs highlighted. (**B** to **D**) Representative images of VeroE6 cells transfected with plasmids encoding the indicated E-mEmerald plasmids and LAMP1-tdTomato (B) with quantification of the overlap assessed by Mander’s correlation coefficient (M2) from 15 imaged cells (C). Data represent M2 coefficients from non–Golgi E–positive regions of each cell, with means ± SD displayed. Tubular E-mEmerald localization (D) was scored visually from 15 imaged cells in three independent experiments, with means ± SE displayed. For (C) and (D), statistical significance determined by one-way ANOVA with Dunnett’s correction for multiple testing. (**E**) Sequence alignment of the cytosolic region of E responsible for Golgi-to-lysosome trafficking and the equivalent region from β- and α-coronaviruses. (**F**) Schematic of E chimeras in which amino acids 56 to 63 were deleted or replaced with equivalent residues from β-coronaviruses (MERS, OC43) or α-coronaviruses (TGEV, 299E). (**G** and **H**) Representative images of VeroE6 cells transfected with the indicated E-mEmerald plasmids (G) and quantification of cells displaying retention of E-mEmerald in Golgi-derived tubules (H) from 10 to 45 imaged cells per experiment across three independent experiments. Mean ± SE is displayed, significance determined by one-way ANOVA with Šidák’s correction for multiple testing. (**I**) Cell lysates and GFP-Trap immunoprecipitations from 293T cells transfected with plasmids encoding GFP or E-mEmerald were resolved by SDS–polyacrylamide gel electrophoresis (SDS-PAGE) and examined by Western blotting with antisera raised against GFP or AP1B1. In microscopy panels, scale bars are 10 μm.

We next returned to our alanine-scanning mutagenesis to explore viral sequences necessary for ARFRP1- and AP-1–dependent Golgi export of E. We noted that E-mEmerald^M5^ was exported from the ER but was not delivered from the Golgi to lysosomes and was retained in GRASP55-positive tubules emanating from this organelle (Fig. 5, A to C, and fig. S2, A to C). We confirmed tubular retention of E-mEmerald^M5^ in A549, Caco-2, and Calu-3 cells (fig. S2E). Adaptins recognize cargos by binding short linear interaction motifs (SLIMs) presented in the cytosolic region of transmembrane cargos. SLIMs including YxxΦ and FxxFxxxR are recognized by hydrophobic pockets in Mu-2 and Beta-2 adaptins, respectively (*41*–*43*). These hydrophobic pockets are well conserved in Mu-1 and Beta-1 adaptins, and we noted similarities between the sequences surrounding the residues mutated in E-mEmerald^M5^ that were necessary for Golgi export and these SLIMs (Fig. 5A). We mutated either Y59A or F56A/Y59A/K63A (E-mEmerald^Y59A^ and E-mEmerald^FYK-AAA^) to disrupt these putative AP-1 interactions and examined lysosomal delivery of E-mEmerald. While E-mEmerald^Y59A^ was delivered normally to lysosomes, we found that E-mEmerald^FYK-AAA^ was retained in Golgi-derived tubular carriers, mimicking the effects of E-mEmerald^M5^ or the effects of inactivating either ARFRP1 or AP-1 (Fig. 5, B to D). The distribution of hydrophobic and basic residues in this region (residues 56 to 63) is well conserved among β-coronaviruses but is absent from α-coronaviruses (Fig. 5E). We deleted this sequence from E-mEmerald and exchanged it with equivalent sequences from either β-coronaviral (MERS-CoV and OC43) or α-coronaviral (hCov299 or TGEV) E proteins (Fig. 5F). Confirming requirements for this region in Golgi export, E-mEmerald^Δ56–63^ localized to Golgi-derived tubules (Fig. 5, G and H). Delivery to peripheral puncta was rescued by insertion of equivalent sequences from β-coronaviral, but not α-coronaviral, E proteins (Fig. 5, G and H). We used GFP-Trap coprecipitation assays to test interaction with AP-1. We found that E-mEmerald could bind HA-tagged and endogenous AP1B1 (Fig. 5I and fig. S10D), that deletion of residues 56 to 63 reduced the interaction with HA-AP1B1, and that this binding could be rescued using chimeric sequences from β-coronaviral, but not α-coronaviral, E proteins (fig. S10, D and E). These data identify an ARFRP1- and AP-1–dependent membrane trafficking pathway that exports E from the Golgi to lysosomes, identify viral sequences that bind AP-1, and demonstrate conservation of these properties among β-coronaviral, but not α-coronaviral, E proteins.

### Lysosomal delivery of E facilitates SARS-CoV-2 replication

**Fig. 6.**
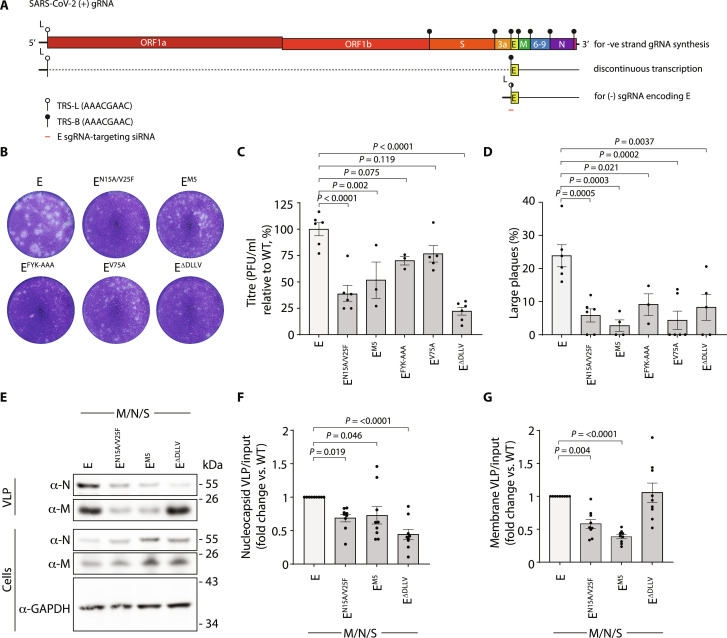
SARS-CoV-2 Envelope trafficking mutants disrupt viral egress. (**A**) Schematic of TRS in the SARS-CoV-2 genome, the discontinuous transcription of sgmRNAs, and the design location of E-sgmRNA siRNAs. (**B**) Supernatants from VeroE6 cells containing the indicated doxycycline-inducible codon-optimized E constructs, which had been transfected with E-sgmRNA–targeting siRNA, treated with doxycycline, and infected with SARS-CoV-2 (hCoV-19/England/02/2020), were used to infect fresh VeroE6 cells, and plaques were allowed to develop for 3 days before being fixed and stained using 0.2% toluidine blue. Images show representative plaque formation. (**C**) Quantification of plaque formation represented as titer (plaque-forming unit/milliliter) using the ViralPlaque FIJI macro. Means ± SE presented with significance calculated by a one-way ANOVA with Dunnett’s correction applied for multiple testing. (**D**) Quantification of the percentage of large plaques versus total plaques from plaque assays. Large plaques were defined by having an area greater than 0.82 mm^2^ and measured using the ViralPlaque macro in FIJI. Means ± SE presented, with significance calculated by a one-way ANOVA with Dunnett’s correction applied for multiple testing. *N* = 3 to 6 independent experiments, as indicated by data points. (**E**) Resolved cell lysates and VLP fractions from 293T cells transfected with the indicated codon-optimized versions of M, N, S, and E were examined by SDS-PAGE and immunoblotted using antisera raised against SARS-CoV M and SARS-CoV-2 N. (**F** and **G**) Quantification of N or M present in VLPs generated using either WT or mutant versions of E normalized against N or M present in cell lysates. Data plotted as fold change relative to WT. Means ± SE presented from *N* = 9, with significance calculated by a one-way ANOVA with Dunnett’s correction applied for multiple testing.

Trans-complementation assays have proved powerful for understanding viral elements necessary for replication in a variety of systems (*44*, *45*). To understand how both ER export and ARFRP1/AP-1–dependent delivery of E from Golgi to lysosomes contributes to the SARS-CoV-2 replication cycle, we developed an RNA interference strategy allowing targeting of the subgenomic SARS-CoV-2 RNA responsible for producing E. Like other *Nidovirales*, SARS-CoV-2 uses discontinuous transcription during negative-strand RNA synthesis to allow template switching between transcription-regulating sequences (TRSs) in the leader sequence of *ORF1A/B* (TRS-L) and identical sequences (TRS-B) immediately upstream of open reading frames (ORFs) in the 3′-end of the genome (Fig. 6A) (*46*–*48*). This allows production of the subgenomic RNAs (sgRNAs) encoding S, E, M, N, and several nonstructural proteins. Using firefly and renilla luciferase reporters, we designed siRNAs targeting the TRS/E junction (fig. S11, A and B) to deplete sgRNA encoding E. We identified sequences that targeted E sgRNA but spared both genomic SARS-CoV-2 RNA and N sgRNA (fig. S11, C and D) and used quantitative reverse transcription polymerase chain reaction (qRT-PCR) to confirm that these oligos were able to target E sg-mRNA, but not N sg-mRNA, in the context of a viral infection (fig. S11E). We next used the *Sleeping Beauty* retrotransposition system (fig. S11F) to integrate a cassette encoding a constitutively expressed tdTomato and a doxycycline-inducible codon-optimized version of E into VeroE6 cells to allow trans-complementation of E. We verified doxycycline-inducible expression of E-mEmerald in equivalently transposed VeroE6 cells sorted on tdTomato (fig. S11G), generated equivalently sorted versions of VeroE6 cells expressing codon-optimized doxycycline-inducible versions of E, E^N15A/V25F^, E^M5^, E^FYK-AAA^, E^V75A^, or E^ΔDLLV^, and transfected them with E-targeting siRNA. After 20 hours, we infected these cells with SARS-CoV-2 (hCoV-19/England/02/2020) and assessed the titer of virus produced via trans-complementation by using plaque assay. As expected, depletion of E attenuated, although did not eliminate, the amount of infectious SARS-CoV-2 produced (fig. S11, H to J). This could be rescued robustly by trans-complementation with WT E but not by versions of E that were retained in the ER (E^V75A^, E^ΔDLLV^) or could not be exported from the Golgi and delivered to lysosomes (E^M5^, E^FYK-AAA^) in the producer cell (Fig. 6, B to D). Trans-complementation with a version of E containing mutations that abrogate its viroporin activity (E^N15A/V25F^) (*49*) did not rescue viral titers in this system (Fig. 6, B to D). These data provide functional evidence that intracellular trafficking of E from both ER-to-Golgi and Golgi-to-lysosomes in host cells supports SARS-CoV-2 replication. Consistent with other systems in which recombinant β-coronaviruses lacking E produce smaller and irregularly shaped plaques (*13*), trans-complemented versions of SARS-CoV-2 bearing versions of E with disrupted viroporin activity, or that were unable to reach lysosomes, produced smaller plaques (Fig. 6D). Last, to distinguish entry and release effects, we turned to a virus-like particle (VLP) system to examine roles for E in particle assembly and release. Using a four-component (E, S, M, and N) SARS-CoV-2 VLP system, we found that particle release was impaired when we used versions of E that either were defective in their viroporin activity (E^N15A/V25F^) or could not be trafficked from Golgi to lysosomes (E^M5^) (Fig. 6, E to G). We also found that particle production was permissible using versions of E that were restricted to the ER (E^ΔDLLV^), but in this case, packaging of N was impaired, suggesting that the site of assembly allows proper biogenesis of SARS-CoV-2 particles (Fig. 6, E to G). These data suggest that trafficking of E as a functional viroporin to lysosomes contributes to late stages of the SARS-CoV-2 replication cycle.

## DISCUSSION

We have demonstrated that the small Envelope protein from SARS-CoV-2 encodes sequence-specific information that enables it to navigate the host’s endomembrane network, allowing its routing to lysosomes where it acts as a viroporin to neutralize the pH in these organelles. We found that E encodes a C-terminal ER-export sequence, mediated primarily by a C-terminal hydrophobic residue that allows engagement with COP-II via SEC24. C-terminal hydrophobic residues are conserved across α, β, and γ coronaviruses, pointing to a conserved mechanism of ER-export for E proteins across *Coronaviridae*. Previously published C-terminally tagged versions of E localize inappropriately to the ER (*50*), and we suggest here that this is due to occlusion of this dominant ER-export sequence.

Second, our internally tagged versions of E allowed us to report that while most E localizes to the Golgi, a pool of E is delivered from here to the lysosomes. We identified sequence motifs within the cytosolic C terminus of E that allow its Golgi-to-endosome trafficking, and we exposed a role for ARFRP1 in coordinating an AP-1–and AP1AR/Gadkin-dependent pathway that allows trafficking of E from Golgi to lysosomes. Of note, AP-1 and AP1AR/Gadkin have been implicated previously in the release of cargo by secretory lysosomes (*30*). ARFRP1 is needed for both the recruitment of golgins and the Golgi Associated Retrograde Protein (GARP) complex to the TGN (*51*), binds AP-1 in a GTP-dependent manner, and controls TGN export of the planar cell polarity protein, Vangl2 (*37*, *52*). We show here that ARFRP1’s GTPase activity is necessary for both AP-1 recruitment to the Golgi and Golgi-to-endosome trafficking of E. AP-1 plays a complex role in bidirectional traffic between the Golgi and endosomes, acting alone in the retrograde pathway from endosomes to Golgi, and in concert with GGAs and AP1AR/Gadkin in the anterograde pathway from Golgi to endosomes (*33*). Consistent with the role of AP-1 in the anterograde movement of E, when this pathway was inactivated, we observed a failure of Golgi export, rather than a redistribution of E to endosomes. We show that the Golgi retention phenotype of E-mEmerald^M5^ is attributable to the loss of AP-1 binding, and we show that sequences required for AP-1 binding and for Golgi-to-endosome trafficking are conserved within β-coronaviruses, but not α-coronaviruses. This region of SARS-CoV E appears to contribute to Golgi retention of a vesicular stomatitis virus glycoprotein/SARS-CoV E chimera (*8*), indicating that AP-1 interaction may prevent this chimera accessing the constitutive secretory pathway. In summary, these data suggest that β-coronaviral E proteins have evolved to exploit an ARFRP1/AP-1–dependent trafficking pathway for transport between Golgi and endosomes, and provide context to the identification of AP-1 in genome-wide screens for host factors regulating SARS-CoV-2 replication.

Consistent with the work of others, and findings in SARS-CoV-2–infected cells (*16*, *17*), we found that E was able to neutralize lysosomal pH. While the ORF3a proteins of SARS-CoV or SARS-CoV-2 have been proposed as ion channels (*53*), recent cryo–electron microscopy and electrophysiological evidence suggests that these proteins do not act as viroporins (*54*) and that they impose their effect on lysosomal biology through interaction with the Homotypic Fusion and Protein Sorting (HOPS) complex (*54*, *55*). Given the potential for viral egress through deacidified secretory lysosomes and the finding that lysosomal pH is neutralized in SARS-CoV-2–infected cells (*15*, *16*), we suggest that trafficking of E to lysosomes contributes to the pH neutralization in this organelle.

What role does lysosomal pH neutralization play in the SARS-CoV-2 life cycle? In SARS-CoV systems, E’s channel activity was necessary for viral pathogenesis, with recombinant viruses bearing channel mutations acquiring compensatory mutations to restore ion flux (*49*). The omicron variant of SARS-CoV-2 encodes a version of E with a point mutation (E^T9I^) in a polar channel-lining residue being less able to neutralize lysosomal pH, which contributes to a reduced viral load in SARS-CoV-2–infected cells (*17*). That the combined channel and oligomerization mutant of E (E^N15A/V25F^) was poorly able to support SARS-CoV-2 replication when supplied in-trans suggests that channel activity is necessary for a productive infection. We reasoned that neutralization of lysosomal pH either would limit exposure of internalized virus to this proteolytic compartment or could protect virions in secretory lysosomes from this degradative environment. Our VLP assays allowed examination of egress effects, and our findings that E^N15A/V25F^ or E^M5^ reduced VLP release suggest that these deacidified lysosomes are important for preserving particles during egress. We also note that our VLP assays also showed that packaging of N was most efficient when E contained an intact C terminus. While in SARS-CoV N has been proposed to interact with the extreme C terminus of E (*56*), the reduced incorporation of N into VLPs containing E^ΔDLLV^ may also be a consequence of restricted localization of E to the assembly site.

Last, our proximal interactomes provide a powerful resource for understanding host factors that may regulate E’s biology. Notably, we recovered many PDZ domain–containing proteins that were biotinylated in a manner requiring E’s extreme C terminus, many of which have been subsequently validated, including PALS1 (*23*) and tight junction protein–1 (TJP1) (*57*) and which likely contribute to epithelial barrier function. Our interactomes differ from those reported by affinity purification (*1*) but do not suffer from high-level overexpression or placement of affinity or BioID tags that would disrupt the normal localization of E (*1*, *25*–*27*). Second, we present a trans-complementation assay allowing depletion and rescue of sgRNAs encoding SARS-CoV-2 structural proteins, allowing us to take reverse genetic approaches without needing to create genetically modified recombinant SARS-CoV-2 viruses. We anticipate that targeting the TRS elements for alternate sgRNAs will allow trans-complementation of these proteins across *Nidovirales*.

In summary, our data have outlined trafficking pathways and routes taken by the E viroporin of SARS-CoV-2, linking viral sequences with cellular factors that govern movement between the ER, Golgi, and lysosomes. We have uncovered pathways responsible for the localization of AP-1 at Golgi membranes. We find specific effects of E on the neutralization of lysosomal pH, which enable efficient particle release and SARS-CoV-2 replication. Besides facilitating viral egress, given the role of the lysosome as a terminal degradative organelle for a variety of cellular routes, we suspect that E’s expression will have wide-ranging effects on the proteostatic capabilities of infected cells.

## MATERIALS AND METHODS

### Cell culture

Short Tandem Repeat (STR)-profiled, mycoplasma-free vials of human embryonic kidney (HEK) 293 (CVCL_0045), 293T (CVCL_0063), A549 (CVCL_0023), Caco-2 (CVCL_0025), Calu-3 (CVCL_6069), and VeroE6 cells (CRL-1586, Pasteur) were obtained from the Crick Cell Services Science Technology Platform. HEK293, 293T, and VeroE6 cells were cultured in Dulbecco’s modified Eagle’s medium (DMEM) containing 10% fetal bovine serum (FBS); A549 cells were cultured in F12 medium containing 10% FBS; Caco-2 cells were cultured in Eagle’s minimum essential medium containing 20% FBS; Calu-3 cells were cultured in Eagle’s minimum essential medium containing 10% FBS. All media were supplemented with penicillin (100 U/ml) and streptomycin (0.1 mg/ml), and all cells were cultured at 37°C and at 5% CO_2_.

### Plasmids

Native sequences corresponding to the alpha variant of SARS-CoV-2 S, N, and E cDNAs were purchased from GenScript Biotech: pUC57-2019-NCov-S MC_0101080, pUC57-2019-nCov-N MC_0101085, and pUC57-2019-nCOV E MC_0101078. Codon-optimized S and N sequences were gifts from N. McDonald (Crick) and were cloned similarly into pCR3.1. A sequence corresponding to the native sequence of M was synthesized by GeneWIZ. Coding sequences were amplified by PCR, and Eco RI–Not I was inserted into pCR3.1 for mammalian expression. An internal Eco RI site in E was removed by silent mutagenesis. Insertion of HT or mEmerald in the E coding sequence was performed using HiFi DNA Assembly, with HT amplified by PCR from pHTN-HT CMV-neo (Promega) and mEmerald amplified by PCR from mEmerald-Sec61b-C1 (Addgene, no. 90992), with a Gly-Gly-Gly-Ser linker placed either side of the HT or mEmerald at site 3 and site 4, a single linker placed between E N/C terminus and HT at tag site 1 or 5, and a Gly-Gly-Gly-Ser-HT-Gly-Gly-Gly-Ser-Glu-Glu inserted at site 2. HA-TurboID tagging at sites 3 and 4 was performed by HiFi DNA Assembly, with HA-TurboID amplified by PCR from 3× HA-TurboID-NLS_pCDNA3 (Addgene, no. 107171) and inserted with a Gly-Gly-Gly-Ser linker either side of the HA-TurboID sequence. Emerald-TurboID was used for a cytosolic control in proximity biotinylation experiments and was generated by using HiFi DNA Assembly to assemble Emerald-TurboID in a pLXIN vector, with mEmerald amplified from mEmerald-Sec61b-C1 (Addgene, no. 90992) and TurboID amplified from 3× HA-TurboID-NLS_pCDNA3, with the assembled construct cut with Age I and Eco RI to replace enhanced GFP (EGFP) in pEGFP-C1 (Addgene, no. 54759) also digested with Age I and Eco RI. pHLARE plasmids were a gift from D. Barber (University of California, San Francisco). HA-Dynamin2^K44A^ was a gift from S. Neil (King’s College London). LAMP1-tdTomato was a gift from M. Gutierrez (The Francis Crick Institute). E mutants were generated by either traditional PCR or two-step PCR depending on mutation position. TGN46-mCherry was expressed from pLVX TGN46-mCherry, a gift from D. Stephens (University of Bristol, Bristol). TGN46-EGFP was a gift from S. Tooze (The Francis Crick Institute). GFP controls were expressed from a pCR3.1 GFP–Eco RI–Xho I–Not I (*58*). A cDNA encoding mCherry flanked by the 18–amino acid signal sequence from BIP (MKLSLVAAMLLLLSAARA) and a C-terminal KDEL sequence was created by PCR and cloned Eco RI–Not I into pMSCVneo–Eco RI–Xho I–Not I. A cDNA encoding ARFRP1 was synthesized by GeneWIZ, and Eco RI/Not I was cloned into pCR3.1. Mutations in pCR3.1 ARFRP1 were generated using PCR. An image clone (OHS5894, clone ID 100000476) encoding human AP1B1 was purchased from Horizon Discovery, and the coding sequence was cloned into pCR3.1 HA–Eco RI–Xho I–Not I using PCR. Envelope 56-63 CoV chimera gene blocks with Eco RI and Not I overhangs were synthesized by GeneWIZ and cloned into pCR3.1 using the Eco RI and Not I digestion cloning described above, with mEmerald inserted into site 3 as previously described. CRISPR knockouts were performed by transfection with a modified version of px330 (Addgene, no. 42230), which encodes Sniper Cas9 (*59*) and EBFP2 joined by a P2A site in place of px330’s original Cas9. Bbs I sites in Sniper-Cas9 (Addgene, no. 42230) were removed by silent mutagenesis, and the “px330-Sniper-P2A-Blue Fluorescence Protein (BFP)” plasmid was created by HiFI DNA Assembly, with the px330 plasmid linearized to remove Cas9 by PCR, mTagBFP2 amplified by PCR from mTagBFP2-C1 plasmid (Addgene, no. 54665), Bbs I–silenced Sniper-Cas9 amplified by PCR, and the P2A synthesized by Integrated DNA Technologies (IDT). To generate px330-Sniper-P2A-BFP (blue fluorescent protein) plasmids for CRISPR knockouts specific for each gene, overlapping oligonucleotides encoding the guide RNA (gRNA) sequence on both parallel and antiparallel strands with Bbs I compatible overhangs were synthesized by IDT, annealed, and ligated into px330-Sniper-P2A-BFP digested with Bbs I. Optimal gRNA designs were selected using CRISPick (Broad Institute) (*60*). For the dual-luciferase reporter for sgmRNA specificity, pRL-TK Envelope sgmRNA, pRL-TK Nucleocaspid sgmRNA, and pGL4–54 Envelope genomic RNA plasmids were used to assess the effectiveness and specificity of E-sgmRNA–targeting siRNAs. pRL-TK plasmids expressed the 5′ untranslated region (5′UTR) of E or N sgmRNA including the TRS-L element, the first 99 nucleotides of the CoV-2 protein from the SARS-CoV-2 genomic sequence, and an in-frame P2A linking to Renilla Luciferase. pGL-54 genomic Envelope plasmids expressed the 5′UTR of Envelope genomic RNA including the TRL-B element, δORF3a, the first 99 nucleotides of Envelope from the SARS-CoV-2 Envelope genomic sequence, and an in-frame P2A sequence linking to Firefly Luciferase. pRL-TK plasmids were constructed by digesting the vector with Nhe I and Hind III and ligation of the 5′mRNAUTR-99ntORF-P2A insert by HiFi DNA Assembly. The pGL4-54 genomic E plasmid was constructed by digesting the vector with Hind III, dephosphorylating using Quick CIP (NEB), and ligation of the 5′ genomicUTR-99ntORF-P2A by HiFi DNA Assembly. pRL-TK and pGL4-54 plasmids were from Promega, and inserts were synthesized by Eurofins. Sleeping Beauty pSBtet-RN (*61*) was a gift from D. Bauer (The Francis Crick Institute). To clone E-Emerald, Emerald, or codon-optimized versions of E mutants, Sleeping Beauty vectors were linearized using PCR at the Direct Repeat (DR) insertion sites, inserts encoding these proteins were synthesized (Eurofins), and the plasmids were assembled using HiFi DNA assembly. SuperPiggyBack hypertransposase was a gift from A. Isaacs (University College London).

### Antibodies and fluorescent labels

An antibody against glyceraldehyde-3-phosphate dehydrogenase (MAB374) was from Millipore; an antibody against SARS-CoV-2 S (GTX632604) was from GeneTex; an antibody against SARS-CoV-2 N (BS-41408R) was from Bioss; an antibody against SARS-CoV M (101-401-A55) was from Rockland; an antibody against ERGIC53 (E1031) was from Sigma-Aldrich; an antibody against GM130 (610822) was from BD Biosciences; an antibody against TGN46 (ab50595) was from Abcam; an antibody against EEA1 (610457) was from BD Biosciences; an antibody against HA.11 (16B12) was from BioLegend; an antibody against HT (G9211) was from Promega; an antibody against GFP (7.1/13.1) was from Roche; an antibody against RER1 (HPA051400) was from Sigma-Aldrich; an antibody against PALS1 (17710-1-AP) was from Proteintech; an antibody against GORAPS2 (10598-1-AP) was from Proteintech; an antibody against ARFRP1 (PA5-50606) was from Invitrogen; an antibody against AP1B1 (16932-1-AP) was from Proteintech; an antibody against AP1G (A4200, clone 100/3) was from Sigma-Aldrich; an antibody against GGA1 (25674-1-AP) was from Proteintech; an antibody against AP1AR (NBP1-90879) was from Novus Biologicals; an antibody against Golgin-97 (PA5-30048) was from Invitrogen; an antibody against GM130 (12480S) was from Cell Signaling Technology; an antibody against GRASP55 (10598-1-AP) was from Proteintech; an antibody against Giantin (sc-46993) was from Santa Cruz Biotechnology; antibodies against LAMP1 were from BD Biosciences (555798) and Abcam (ab24170); an antibody against CD63 (H5C6-S) was from DSHB; an antibody against SARS-CoV-2 N (DA114) was from MRC PPU University of Dundee (*62*); an antibody against annexin V conjugated to Allophycocyanin (APC) (640932) was from BioLegend; horseradish peroxidase (HRP)–conjugated streptavidin (S911) was from Invitrogen; Alexa-conjugated secondary antibodies were from Invitrogen; and HRP-conjugated secondary antibodies were from Millipore. IRDye 800 CW (925-32210) and IRDye 680 RD (925-68071) were from LI-COR Biosciences. Alexa Fluor 647–conjugated transferrin was from Molecular Probes. Janelia Fluor 646 (JF646) HT ligand (GA1120), Oregon Green HT ligand (G2801), and TMR HT ligand (G8251) were from Promega.

### Transient transfection of cDNA

VeroE6 cells were transfected using Lipofectamine 3000 (Life Technologies) according to the manufacturer’s instructions. 293T cells were transfected using linear 25-kDa polyethylenimine (PEI; Polysciences Inc.), as described previously (*63*).

### siRNA depletion

All siRNA-based depletions were performed at 20 nM final concentration using Lipofectamine RNAiMAX (Life Technologies) transfection reagent according to the manufacturer’s instructions. AP1M1 (L-013196-00-0005), AP1AR (L-015504-02-0005), and GGA1 (M-013694-01-0005) were depleted using ON-TargetPlus SMARTpool siRNAs (Horizon Discovery). A range of custom-made siRNAs (data S5) were synthesized by Horizon Discovery to specifically deplete SARS-CoV-2 E sg-mRNA. These siRNAs were of different lengths and spanned E’s 5′UTR and ORF.

### Fixed-cell imaging

VeroE6 cells were plated at 40,000 per well on 13-mm No. 1.5 coverslips and transfected the following day as described. If cells were transfected with HT versions of SARS-CoV-2 E, cells were treated with 1 μM Oregon Green Halo ligand for 20 min and then washed three times with complete media, with a 5- to 10-min incubation on the final wash. All cells were washed once with phosphate-buffered saline (PBS) before being fixed using 4% paraformaldehyde (PFA) for 20 min. Cells that required immunolabeling were permeabilized with 0.1% Triton X-100 in PBS, washed three times in PBS, and blocked in 5% FBS for 1 hour. For LAMP1 immunostaining, cells were permeabilized with PBS supplemented with 0.5% saponin, and saponin was included at 0.1% in all subsequent wash and antibody incubation steps. Lysosome size was calculated as described previously (*64*). After primary and secondary antibody incubations, coverslips were mounted on X50 SuperFrost microscope slides using Mowiol. Imaging was performed using either a Zeiss LSM 880 as described below or an Andor Dragonfly 200 spinning disc confocal paired with a Zyla 5.5 sCMOS camera and using a Nikon Eclipse Ti2 with Plan Apo 60×/1.4 numerical aperture (NA) or 100×/1.45 NA objectives. To limit overexpression, the cells were fixed or imaged 16 to 18 hours after transfection. Representative images displayed in the figures were acquired on Zeiss LSM 880; E phenotype quantification was performed on 50 cells per condition, acquired on Dragonfly 200, with sample identification randomized and blinded during scoring.

### Live-cell imaging

Cells stably expressing the indicated proteins, or edited to express fluorescent proteins, were plated in four- or eight-chamber slides (Ibidi). VeroE6 cells were plated at 40,000 per well in μ-slide ibiTreat four-well Ibidi chambers and transfected the following day, as described. After 16 to 18 hours, if required, the cells were treated with 200 nM JF646 Halo ligand in complete media for 20 min and were then washed twice in growth media before being imaged in FluoroBright DMEM supplemented with 10% FBS, 4 mM l-glutamine, penicillin (100 U/ml), and streptomycin (0.1 mg/ml). Airyscan imaging was performed using a Zeiss LSM 880 inverted microscope with a Plan Apo 63×/1.4 NA objective fitted with a Fast Live Cell Airyscan detector, definite focus, and heat and CO_2_ incubation. Acquired images were processed using Zeiss’ “Auto” 2D Airyscan processing, and image brightness levels and image crops were adjusted and performed using the FIJI distribution of ImageJ. To limit overexpression, the cells were imaged 16 to 18 hours after transfection.

### FLIM-FRET imaging

VeroE6 cells were transfected as previously described with E-mEmerald as the fluorescence donor and either empty vector for a single color control or E-HT mutant illuminated with TMR HT ligand for the fluorescence acceptor. A 1:3 ratio was used for fluorescence donor to acceptor. The TMR HT ligand (5 μM) was applied to cells for 30 min before the cells were washed three times in growth media and then incubated for 30 min before the cells were imaged in live-cell imaging media. FLIM imaging was performed on a Leica TCS SP8 Multiphoton FALCON with an HC PL APO CS2 63×/1.40 oil objective using 470- and 552-nm laser lines at 100-Hz scan speed scanning by line, with samples incubated at 37°C and in 5% CO_2_. Time-correlated single-photon counting fluorescence lifetime data were acquired using a PicoQuant PDL 800-D unit. Raw files were then exported into FLIMfit software (*65*) for analysis. Intensity images for each FLIM image were exported into FIJI, and a custom script was written to segment each lysosome, allowing the fluorescence lifetime of each lysosome to be calculated individually in FLIMfit. The Golgi was excluded from this analysis. For all FLIM analysis, no binning was used, and a single exponential curve was fitted to the data to calculate fluorescence lifetime on a pixel-wise basis.

### Transferrin internalization assay

VeroE6 cells plated on coverslips were transfected with equivalent amounts of mEmerald-tagged E and either empty plasmid or dominant-negative HA-Dynamin2^K44A^. After 16 hours, the cells were treated with transferrin-647 (T23366) purchased from Thermo Fisher Scientific at 10 μg/ml, resuspended in growth media for 2 min before being washed once in ice-cold PBS, and fixed immediately in 4% PFA. Untreated cells (0 min) were fixed without being treated with transferrin-647. The cells were prepared for fixed-cell imaging, with the presence of HA-Dynamin2^K44A^ detected by an HA antibody. The number of transferrin and E puncta was analyzed in FIJI using a custom-written script to isolate the lysosomal puncta, with these counts corrected for differing cell area.

### “Quilt” quantification of subcellular distribution

A heatmap-based approach was devised to provide a quickly interpretable graphical display of subcellular localization across multiple organelles in imaging datasets [quantitative imaging-based localization table (QUILT)]. This approach was used to highlight the variability and dominant distributions of E in the secretory pathway and score the perinuclear distribution of AP1 but could be adapted for other classifications of subcellular distribution. To generate a quilt, each imaged cell (>50) was scored for strength of localization of E in the ER, the Golgi, in punctae, at the plasma membrane (not shown), or having a perinuclear distribution in the case of AP-1. For ER, Golgi, and perinuclear AP-1 distributions, “None” was defined by fluorescent images having no reticular or Golgi/AP-1 perinuclear pattern visible, “Few” was defined by only a minority of signal being reticular ER or Golgi/AP1 perinuclear relative to the rest of the distribution of the fluorescence, and “Strong” was defined by the reticular ER or perinuclear Golgi/AP-1 fluorescence being highest or equal highest fluorescence in the image. For punctate localization, these were scored as None, Few, and “Many,” with this quantification judged subjectively relative to the total cell size. Few typically corresponded to <0.045 puncta/μm^2^, and Many typically corresponded to >0.045 puncta/μm^2^. No distinction was made between different types of puncta. Golgi was defined by perinuclear fluorescence, and the ER was defined by a reticular morphology and nuclear envelope localization. Once scored, the totals for each compartment category were summated across all the cells imaged in each condition, converted to a percentage of the total number of cells in that condition, and plotted as a heatmap using R.

### Apoptosis assay

Apoptosis was analyzed using the BioLegend APC Annexin V Apoptosis Detection Kit with propidium iodide (PI; 640932). Cells were plated in 24-well plates at 60,000 cells per well and transfected the following morning with either E-mEmerald^Site3^ or GFP. After either 16, 24, 48, or 72 hours, the cells were trypsinized, neutralized in complete media, centrifuged, and washed twice in 5 ml of PBS. After the second wash, the cell pellets were resuspended in 100 μl of annexin V binding buffer and mixed with 5 μl of annexin V–APC antibody and 10 μl of PI and incubated at room temperature for 15 min. After this time, 400 μl of annexin V binding buffer was added, and the samples were analyzed on a Beckman Coulter CytoFLEX LX flow cytometer. GFP/Emerald-positive cells were detected using a 488-nm laser and a 525-40 band-pass filter, annexin V–APC was detected using a 638-nm laser and a 660-10 band-pass filter, and PI was detected using a blue laser and a 690-50 band-pass filter. At least 5000 GFP/Emerald events were acquired per sample, and the percentage of GFP^+^ annexin V–APC^+^ events reported discounting events that were strongly PI^+^ as dead cells.

### pHLARE assay

VeroE6 cells were transfected with E-HT and pHLARE plasmids at equal amounts, and after 16 hours, the cells were incubated with JF646 Halo ligand and imaged by live-cell microscopy on a Zeiss LSM 880 inverted microscope with green, red, and far-red channels scanning by line. Images were analyzed in FIJI using a custom-written script that removed background from all channels, identified lysosomes by their presence in the mCherry red channel, classified these as either E-high or E-low/absent, and then measured the integrated density in the 488 and 561 channels. E-high/low lysosomes were determined by eye and equated to at least a 10-fold difference between the mean of the mean gray intensities of the E-high and E-low lysosomes. The ratios between the 488 and 561 channels for E-high and E-low/absent for each lysosome were then averaged across the cell and averaged across the total number of cells recorded. For controls, VeroE6 cells were transfected with pHLARE plasmids alone and then, after 16 hours, were treated with either 200 nM bafilomycin A1 (19-148) from Sigma-Aldrich for 150 min, 10 μM chloroquine diphosphate (C6628) from Sigma-Aldrich for 160 min, or 10 mM ammonium chloride (254134) from Sigma-Aldrich for 180 min or were untreated; imaged in green and red channels using the system described; and analyzed as described above. Data were collated as described above for E-HT experiments.

### 4-PBA assay

VeroE6 cells were transfected with Halo-tagged SARS-CoV-2 E plasmids for 16 hours. After 16 hours, cells were washed with PBS, incubated with 1 μM Oregon Green Halo ligand for 20 min, and washed six times in media, before being incubated with either H_2_O (“vehicle”), 5, 10, or 20 sodium phenylbutyrate (4-PBA) dissolved in H_2_O for 6 hours. After 6 hours, media containing 5 μM TMR Halo ligand supplemented with the appropriate concentration of 4-PBA were added to the cells for 20 min. The cells were washed three times in media before being fixed and prepared for imaging (as previously described). Sodium phenylbutyrate was from Sigma-Aldrich (SML0309).

### TurboID proximity biotinylation, capture, and MS

HEK293T cells were grown for 5 days in biotin-free growth media to remove all sources of biotin. These were then transferred to T75 flasks, with three flasks seeded per condition. Cells were transfected with either WT or mutant E-HA/TurboID or TurboID (cytosolic control) constructs using PEI, with 1800 μl of Opti-MEM, 36 μg of DNA, and 72 μl of polyethyleneimine (PEI) used per flask. After exactly 18 hours, cells were biotinylated by incubation in biotin-free growth media supplemented with 50 μM biotin (Sigma-Aldrich). After exactly 20 min, flasks were placed on ice and washed once with ice-cold PBS to halt the biotinylation reaction. Cells were scrapped into 10 ml of ice-cold PBS and pelleted, with pellets kept on ice until all samples had been prepared. Cell pellets were lysed by 30-min incubation at 4°C in 1 ml of radioimmunoprecipitation assay (RIPA) buffer [150 mM NaCl, 50 mM tris-HCl (pH 8), 1% NP-40, 0.5% sodium deoxycholate, 0.4% SDS, and 1 mM EDTA] supplemented with cOmplete EDTA-free protease inhibitors (Roche) and benzonase nuclease (167 U/ml, Sigma-Aldrich). During this time, preacetylated NeutrAvidin agarose beads (Pierce) were washed four times with 10× their volume in lysis buffer, with 40 μl of beads used per sample. NeutrAvidin bead acetylation was necessary to stop the NeutrAvidin being cleaved from the agarose beads during on-bead digestion and performed before the day of pulldown by two 30-min incubations of beads with 10 mM sulfo–*N*-hydroxysuccinimide acetate (Thermo Fisher Scientific) on a rotating wheel followed by quenching in 90 mM tris-HCl (pH 7.5). Lysed cell pellets were centrifuged at 28,000*g* at 4°C for 15 min to sediment undigested nuclear debris, and supernatants were mixed with equal amounts of washed acetylated NeutrAvidin beads and rotated at room temperature for 2 hours. The beads were then washed three times in 500 μl of RIPA buffer and six times in 25 mM Hepes (pH 8.5), with the beads rotated for 3 min at room temperature for each wash. After the final wash, beads were resuspended in 100 μl of 25 mM Hepes (pH 8.5), and 100 ng of lysyl endopeptidase LysC (WAKO) was added to each sample, with this mixture incubated for 16 hours at 37°C in a hooded ThermoMixer at 1200 rpm. Each bead supernatant was then transferred to a new Eppendorf and mixed with 100 ng of trypsin (Pierce) and incubated at 37°C for 6 hours. The solutions were then acidified to a final concentration of 0.5% trifluoroacetic acid. Digested samples were loaded onto Evotips and washed once with aqueous acidic buffer (0.1% formic acid in water) before loading onto an Evosep One system coupled to an Orbitrap Fusion Lumos (Thermo Fisher Scientific). The Evosep One was fitted with a 15-cm column (PepSep), and a predefined gradient for a 44-min method was used. The Orbitrap Lumos was operated in data-dependent mode (1-s cycle time), acquiring higher-energy-collisional-dissociation with ion trap (HCD-IT) tandem mass spectrometry (MS/MS) scans in rapid mode after an OT MS1 survey scan (*R* = 60,000). The MS1 target was 4E5 ions, whereas the MS2 target was 1E4 ions. The maximum ion injection time used for MS2 scans was 300 ms, the HCD-normalized collision energy was set at 32, and the dynamic exclusion was set at 15 s. Acquired raw files were processed with MaxQuant v1.5.2.8 (*66*). Peptides were identified from the MS/MS spectra searched against *Homo sapiens* and SARS-CoV-2 proteomes (UniProt) as well as *Gallus gallus* Avidin (UniProt) and sequences of all TurboID-tagged constructs using Andromeda (*67*) search engine. Methionine oxidation, acetyl (N-term), acetyl (K), and deamidation (NQ) were selected as variable modifications. The enzyme specificity was set to trypsin with a maximum of two missed cleavages. The precursor mass tolerance was set to 20 parts per million (ppm) for the first search (used for mass re-calibration) and to 4.5 ppm for the main search. The datasets were filtered on posterior error probability to achieve a 1% false discovery rate (FDR) on protein, peptide, and site level. Other parameters were used as preset in the software. “Unique and razor peptides” mode was selected to allow identification and quantification of proteins in groups (razor peptides are uniquely assigned to protein groups and not to individual proteins). Intensity-based absolute quantification (iBAQ) in MaxQuant was performed using a built-in quantification algorithm (*66*) enabling the “Match between runs” option (time window, 0.7 min) within replicates. MaxQuant output files were processed with Perseus v1.4.0.2 (*68*). Data were filtered to remove contaminants, protein IDs originating from reverse decoy sequences and only identified by site. iBAQ intensities were log_2_-transformed, normalized by median subtraction, and filtered for the presence of 15 valid values. Missing values were imputed from normal distributions. *P* values were calculated by two-sample *t* tests using Benjamini-Hochberg FDR correction for multiple testing. Crapome data were obtained from Crapome V2 (*69*). For quality control, four aliquots were taken during sample processing for each sample: “Input,” 50 μl of lysate before mixing with beads; “Supernatant,” 50 μl of lysate after pull down and pelleting beads; “LysC^−^,” 10 μl of a 100-μl bead resuspension after pull down and bead washing before LysC incubation; “LysC^+^,” 10 μl of a 100-μl bead resuspension after LysC incubation and removal of supernatant containing proteins cleaved from beads. All QC samples were mixed with 4 × lithium dodecyl sulfate (LDS) with β-mercaptoethanol. Input and Supernatant QC samples were run on SDS–polyacrylamide gel electrophoresis (SDS-PAGE) gels, blotted, and incubated with strepatividin-HRP, and LysC^−^ and ‘LysC^+^ samples were run on SDS-PAGE gels and proteins were detected by silver stain using Invitrogen’s SilverQuest staining kit (45-100). All MS proteomics data have been deposited to the ProteomeXchange Consortium via the partner repository with the dataset identifier PXD045299. In the depository, site 3 and site 4 are represented by “TAL” and “VNVS,” respectively, and the data include R61A K63A mutants that have been excluded from the final manuscript. Tabular depiction of LFQ data from all experiments here is provided in data S1 to S3.

### Immunoprecipitation assay

For verification of proximity biotinylation MS data, 25 million 293T cells in a 150-mm dish were transfected with 40 μg of pCR3.1 E-HT^Site3^ or pCR3.1 using PEI. After 48 hours, the cells were rinsed briefly in ice-cold PBS, lifted from the dish using a cell scraper, collected by centrifugation at 300*g*, and lysed on ice in 1 ml of HNG buffer [50 mM Hepes (pH 7.5), 150 mM NaCl, 1 mM EDTA, and 1% glycerol] supplemented with 0.5% digitonin, protease inhibitors (Complete mini), and phosphatase inhibitors (PhosStop). Lysis was performed in low-bind microfuge tubes (Eppendorf). Insoluble material was removed by centrifugation at 14,000*g* for 2 min, and the supernatant was incubated with 50 μl of HNG-washed agarose beads (Chromotek) with end-over-end rotation for 15 min to capture nonspecific binding proteins. Beads were collected by centrifugation and discarded. The supernatant was incubated with 50 μl of HNG-washed HaloTrap-agarose beads (Chromotek) with end-over-end rotation for 15 minutes to capture specific binding proteins. Beads were washed three times in HNG buffer and transferred to fresh tubes. Bead-bound proteins were released by boiling in 2× LDS sample buffer, and samples were analyzed using SDS-PAGE and immunoblotting. To investigate the interaction between Envelope or Envelope mutants and HA-tagged AP1B1, 9 million 293T cells were plated in T75 and transfected the following morning with either 18 μg of pCR3.1 E-mEmerald plasmids or 360 ng of pCR3.1 GFP, 17.6 μg of pCR3.1, and 4.5 μg of pCR3.1 HA-AP1B1 as appropriate. To investigate the interaction between Envelope and endogenous AP1B1, 9 million 293T cells were plated in T75 and transfected the following morning with either 18 μg of pCR3.1 E-mEmerald or 360 ng of pCR3.1 GFP and 17.6 μg of pCR3.1. For all Envelope-AP1 interaction assays, cells were rinsed briefly in ice-cold PBS, lifted using a cell scraper, collected by centrifugation at 300*g*, and lysed on ice in 1 ml of HNG buffer supplemented with 1% NP-40, protease inhibitors, and phosphatase inhibitors. Lysates were rotated for 30 min at 4°C, and insoluble material was removed by centrifugation at 14,000 rpm for 15 min at 4°C. Supernatants were added to 15 μl of GFPTrap-magnetic agarose beads (Chromotek) and incubated with end-over-end rotation for 30 min at 4°C to capture specific binding proteins. Beads were washed five times in HNG buffer supplemented with 0.1% NP-40 with 1 min of end-over-end rotation for each wash. Samples were eluted from beads by incubation at 95°C with 40 μl of 2× LDS sample buffer containing β-mercaptoethanol for 5 min and analyzed using SDS-PAGE and immunoblotting.

### CRISPR-KO and validation

VeroE6 cells were transfected with px330-Sniper-P2A-BFP plasmids each cloned to express a gRNA specific for each gene of interest. After 2 days, the cells were single-cell sorted using a FACSAria Fusion flow cytometer (BD Biosciences) into 96-well plates enriching for BFP-positive populations. Twenty clones from each plate were expanded, and genomic DNA was extracted by a GeneJet Genomic DNA Purification Kit (Thermo Fisher Scientific, K0722) following the manufacturer’s protocol. PCR was then used to generate amplicons of between 150 and 400 nucleotides that surrounded the gRNA annealing region and protospacer adjacent motif (PAM) of the gene of interest, with forward primers designed with a TCGTCGGCAGCGTCAGATGTGTATAAGAGACAG overhang and reverse primers with a GTCTCGTGGGCTCGGAGATGTGTATAAGAGACAG overhang encoding adaptors for Nextera XT Indexing (Illumina). Primers are listed in data S6. PCR products were purified using Ampure XP beads (Beckman Coulter) following the manufacturer’s instructions, with the size of the product verified by agarose gel electrophoresis. Nextera XT Indexing primers were then used to index amplicons by PCR, and the product was purified by Ampure XP beads. Samples were then sequenced using a MiSqeq (Illumina), and alignment of next-generation sequencing results to the genome was performed using SerialCloner software with allele-specific reads reported at approximately a 50:50 ratio. Homozygous RER1 knockout and ARFRP1 knockout were validated by immunoblotting.

### Luciferase assay for envelope sg-mRNA siRNA screening

Luciferase screening of Envelope subgenomic siRNA potency and specificity was performed using the Dual-Luciferase Reporter Assay System (Promega, E19190). For initial screening of siRNA potency, HEK293 cells were plated in 24-well plates at 50,000 cells per well and, after 4 hours, were transfected with 20 μM custom E-sgmRNA–targeting siRNAs or a scrambled control siRNA (data S5). After 24 hours, cells were transfected with a 1:1 ratio of pRL-TK Envelope subgenomic and pGL4-54 Envelope genomic plasmids, with transfection media changed 6 hours after transfection. After 24 hours, the cells were washed once in PBS and lysed by addition of 100 μl of 1× Passive Lysis Buffer (Promega) and incubated on a tabletop rocker for 15 min at room temperature. Lysate was then collected, 20 μl was mixed with 100 μl of LARII reagent, and Firefly Luciferase luminescence was measured by a Promega Glomax 20/20 Luminometer. One hundred microliters of Stop & Glo reagent was then added, and the Renilla Luciferase luminescence was measured. siRNA potency was determined by the fold-change increase of the Luciferase:Renilla ratio compared to a control of a nontargeting siRNA. The top 10 performing siRNAs were then validated for potency and specificity in VeroE6 cells. VeroE6 cells were plated and treated with siRNA as described above. Cells were then transfected with either a 1:1 ratio of pRL-TK Envelope sgmRNA plasmid and pGL4-54 Envelope genomic plasmid or pRL-TK N sgmRNA plasmid and pGL4-54 Envelope genomic plasmid, and measurements of Firefly and Renilla luminescence were measured as before. siRNA potency was assessed as described previously, and siRNA specificity was determined by the fold change of the siRNA’s E^genomic^/E^subgenomic^ ratio against a nontargeting control compared to a E^genomic^/N^subgenomic^ ratio against a nontargeting control.

### Viruses and infection

The SARS-CoV-2 isolate used (hCoV-19/England/02/2020) was obtained from the Respiratory Virus Unit, Public Health England, UK. Virus stocks were propagated in Vero V1 cells (a gift from S. Goodbourn, St George’s University of London) by infection at a multiplicity of infection (MOI) of 0.0001 in DMEM, supplemented with 2% fetal calf serum (FCS) and penicillin-streptomycin (100 U/ml each), and harvested after 4 days. Stocks were titrated on Vero E6 cells (Pasteur). Vero cells were transfected in 24-well plates with 10 pmol of siRNA using Lipofectamine 3000 (Invitrogen). After 2 hours, the medium was replaced with 10% FCS DMEM containing doxycycline hydrochloride (0.5 μg/ml, Thermo Fisher Scientific). After 20 hours, cells were infected with SARS-CoV-2 at a multiplicity of 1 plaque-forming unit (PFU) per cell, in DMEM containing 2% FCS and DEAE-dextran (50 μg/ml). After 2 hours, the inoculum was replaced with 2% FCS DMEM containing doxycycline hydrochloride (0.5 μg/ml) and cells were transfected again with 10 pmol of siRNAs to ensure maximal knockdown. Cells were incubated at 37°C for 24 hours before supernatants were harvested for plaque assay, and cells were harvested in TRIzol (Invitrogen) for quantitative PCR (qPCR) analysis. For trans-complementation assays, bearing the sleeping beauty system, tdTomato-positive cells were obtained by fluorescence-activated cell sorting as described below. For verification of E-Emerald’s localization upon SARS-CoV-2 infection, VeroE6 cells were plated and transfected with E-Emerald; after 48 hours, the cells were infected at an MOI of 1 in DMEM with 2% FCS and penicillin-streptomycin (100 U/ml each) and fixed 18 hours after infection. Cells expressing low levels of E-Emerald were selected for imaging.

### Plaque assay

Confluent VeroE6 cells were infected with diluted supernatants for 30 min. Overlay medium [1× minimum essential medium, 1.2% Avicel, and penicillin-streptomycin (100 U/ml each)] was added, and cells were incubated at 37°C for 3 days. Cells were fixed with 4% PFA in PBS and stained using 0.2% toluidine blue (Sigma-Aldrich). Plaque area was determined using the ViralPlaque macro in FIJI (*70*). Large plaques were defined by having an area greater than 0.82 mm^2^ and measured using the ViralPlaque macro in FIJI (*70*).

### VLP production assay

293T cells (7.5 million) in a 100-mm dish or T75 were transfected with a mixture comprising 5 μg of pCR3.1 SARS-CoV-2 S, 3 μg of pCR3.1 SARS-CoV-2 M, 3 μg of pCR3.1 SARS-CoV-2 E (or derivatives), and 1 μg of pCR3.1 SARS-CoV-2 N. Codon-optimized sequences were used in all cases. Medium was changed after 6 hours. Forty-eight hours after transfection, supernatants were clarified by centrifugation (300*g*, 2 min) and passed through a 0.45-μm syringe filter. Supernatants were underlaid with a PBS–20% sucrose cushion and subjected to ultracentrifugation in a Beckman SW41 Ti swinging bucket rotor at 28,000 rpm for 3 hours at 4°C. Supernatants were removed, and pellets were resuspended in 30 μl of PBS and incubated overnight at 4°C. The next morning, 30 μl of 2× LDS sample buffer was added for sample recovery. Cellular fractions were obtained by lifting cells with PBS and collecting them by centrifugation (300*g*, 2 min) before resuspending the pellet in fresh PBS and adding an equal volume of 2× LDS sample buffer.

### RNA extraction and qPCR

RNA was extracted using the Direct-Zol miniprep kit (Zymo). cDNA was synthesized using Superscript VILO Master Mix (Invitrogen). SARS-CoV-2 ORF1ab and actin were quantified using the 2019-nCoV: Real-Time Fluorescent RT-PCR kit (BGI). E and N sgmRNAs were quantified using specific primer probe sets from (*48*) (sequences below), using TaqMan Multiplex Master Mix (Applied Biosystems). Viral gene expression was normalized to actin expression and expressed as a fold change compared to the scrambled siRNA control cells.

**Table:** 

E-FWD	gtaacaaaccaaccaactttcg
E-REV	ctagcaagaataccacgaaagc
E-Probe	agatctgttctctaaacgaacttatgtactcattcgtt
N-FWD	gtaacaaaccaaccaactttcg
N-REV	ggttactgccagttgaatctg
N-Probe	tgtagatctgttctctaaacgaacaaactaaaatgtct

### Sleeping Beauty generation and flow cytometry

Sleeping Beauty plasmids (pSBtet-RN) expressing codon-optimized versions of E, E-Emerald, or GFP were cloned as previously described and transfected into VeroE6 cells at a 1:1 ratio with the SuperPiggyBac transposase using Lipofectamine 3000. Cells were selected with G418 for 1 week before being grown in normal DMEM. Because of the low genomic transposition efficiency, three rounds of flow cytometry enrichment were performed to achieve high proportions of transposed cells. An E-mEmerald line was generated to compare the level of constitutive tdTomato expression that corresponded to doxycycline-inducible E or E-mEmerald expression. This was determined by treatment of Sleeping Beauty E-mEmerald cells with and without doxycycline hydrochloride (0.5 μg/ml) for 20 hours and assaying for mEmerald-positive cells (detected using a 488-nm laser and a 525-40 band-pass filter) and tdTomato-positive cells (detected using a 561-nm laser and a 610-20 band-pass filter. The brightness of the appropriate tdTomato population was determined by using eight-peak fluorescent beads, allowing the brightness of this population to be quantified. Enrichment of tdTomato-positive populations of an equivalent brightness was performed on a BD FACSAria Fusion flow cytometry sorter gating on the appropriate tdTomato-positive population as determined by acquisition using a 561-nm laser and a 610-20 band-pass filter and plotting against acquisition using a 488-nm laser and a 530-30 band-pass filter to discount autofluorescence. Three rounds of enrichment sorting were performed to enrich the appropriate tdTomato population to >90% before use in infectivity assays.

### Sequence alignments

Alignments of coronavirus Envelope sequences were performed using T-Coffee (*71*). Aligned sequences were then exported and viewed in Jalview (*72*), and residues were color-coded using ClustalX color map.

### Gene ontology analysis

Gene ontology cellular compartment (GO:CC) data were used to categorize the subcellular distribution of proteins identified from proteomic analysis by filtering the list of proteins by these classes. The GO:CC terms used were ER: GO:0005783, ERGIC: GO:0005793, Golgi: GO:0005794, Lysosomal: GO:0005768 (endosome), and GO:0005764 (lysosome).

### Statistical analysis

Two-tailed Student’s *t* tests or ordinary one- or two-way analysis of variance (ANOVA) with the indicated corrections for multiple testing was used to assess significance between test samples and controls and was performed using GraphPad Prism.

## Supplementary Materials

### This PDF file includes:

Figs. S1 to S11 Legends for data S1 to S6 Uncropped Western blots
