# Supplementary material for: ER-export and ARFRP1/AP-1–dependent delivery of SARS-CoV-2 Envelope to lysosomes controls late stages of viral replication
Source: Sci Adv. 2024 Apr 3;10(14):eadl5012. doi: 10.1126/sciadv.adl5012 (PMC10990277; doi:10.1126/sciadv.adl5012)
Supplement: Supplementary file 1 — Figs. S1 to S11 Legends for data S1 to S6 Uncropped Western blots [file sciadv.adl5012_sm.pdf]

Science Advances
AAAS

Supplementary Materials for

ER-export and ARFRP1/AP-1–dependent delivery of SARS-CoV-2 Envelope to lysosomes controls late stages of viral replication

Guy J. Pearson *et al.*

Corresponding author: Jeremy G. Carlton, jeremy.carlton@kcl.ac.uk

Sci. Adv. **10**, eadl5012 (2024)
DOI: 10.1126/sciadv.adl5012

The PDF file includes:

Figs. S1 to S11
Legends for data S1 to S6
Uncropped Western blots

Other Supplementary Material for this manuscript includes the following:

Data S1 to S6

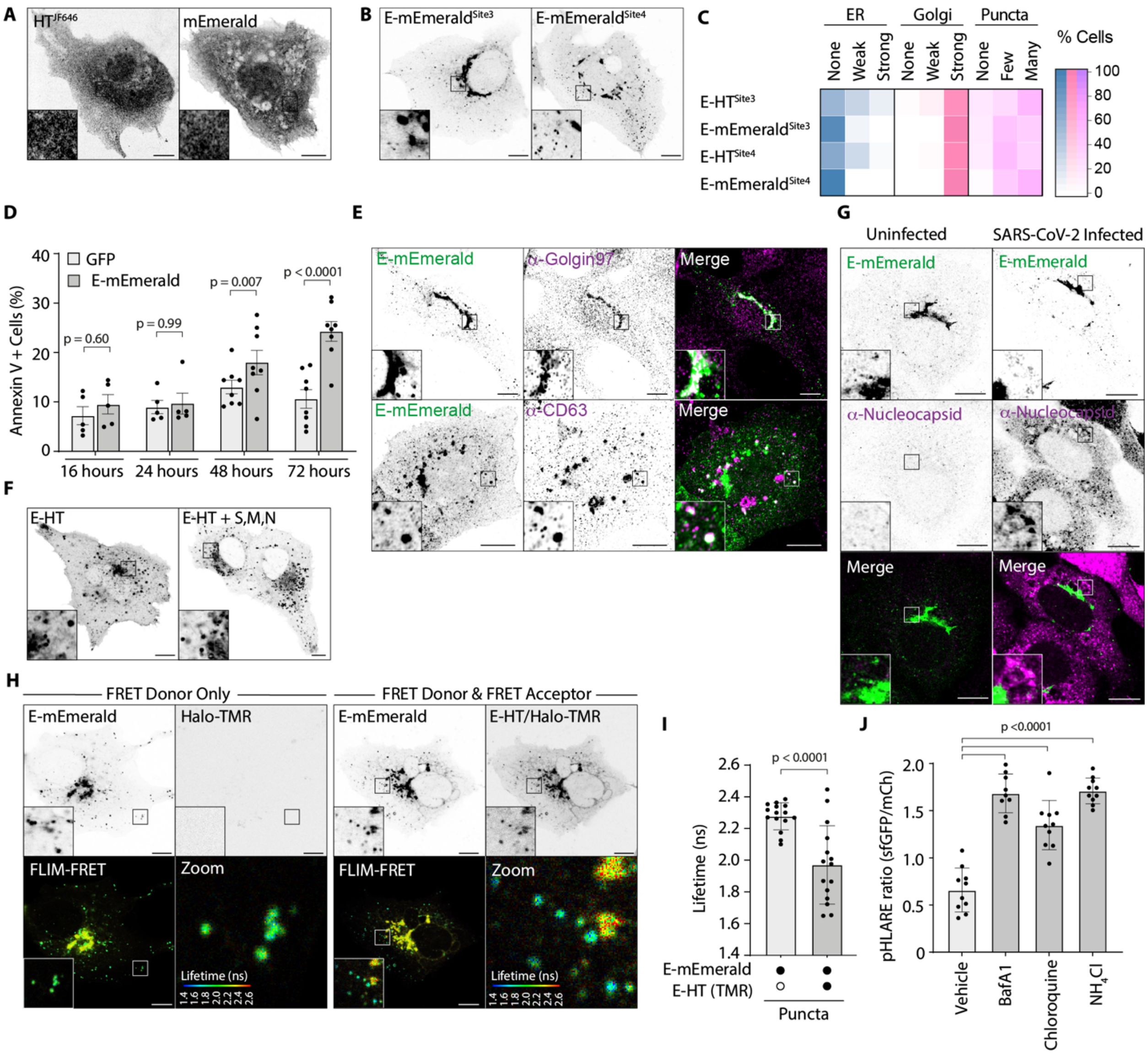

Fig. S1. Validation of tagging strategy and ability of tagged versions of E to deacidify lysosomes. (A, B) Representative images of VeroE6 cells transfected with plasmids encoding mEmerald, HaloTag or the indicated E-mEmerald fusions and imaged live. Images representative of 12 or 8 imaged cells (A), or 15 imaged cells (B). (**C**) Quilt of subcellular distribution of the indicated E-mEmerald or JF646-illuminated E-HT fusions from 50 imaged cells. (**D**) Flow cytometry-based analysis of Annexin V+ VeroE6 cells transfected with either GFP or E-mEmerald for the indicated times, > 3000 GFP+ events captured per condition, N = 5 (16 hours, 24 hours), N = 8 (48 hours and 72 hours). Mean ± S.E. displayed, significance determined by 2-way ANOVA with Šidák's correction. (**E**) Images of VeroE6 cells transfected with E-mEmerald and stained with antisera raised against Golgin97 or CD63. Images representative of 5 or 10 imaged cells per condition. (**F**) Images of JF646-illuminated E-HT fusions transfected into VeroE6 cells either alone or with plasmids encoding SARS-CoV-2 Spike (S), Nucleocapsid (N) and Membrane (M). Images representative of 9 or 15 imaged cells respectively. (**G**) Images of VeroE6 cells transfected with E-mEmerald, infected with SARS-CoV-2 at MOI of 1 for 18 hours, fixed and stained with antisera raised against N. Images representative of 10 imaged cells per condition. (**H**) Representative confocal and FLIM-FRET images of VeroE6 cells transfected with plasmids encoding E-mEmerald and TMR-illuminated E-HT; mEmerald lifetime displayed with rainbow LUT. (**I**) Quantification of E-mEmerald lifetime in segmented punta. Mean ± S.D. displayed, significance testing performed by 2-tailed T-Test from > 40 puncta per cell in 15 imaged cells per condition. (**J**) pHLARE signal from VeroE6 cells transfected with pHLARE and

treated with the indicated compounds (Bafilomycin A1, 200 nM, 150 minutes; Chloroquine, 10 μM, 160 minutes; Ammonium Chloride, 10 mM, 180 minutes). Mean ± S.D. from N = 10 cells per condition, significance calculated by 1-way ANOVA with Dunnett's correction. In microscopy panels, scale bars are 10 μm.

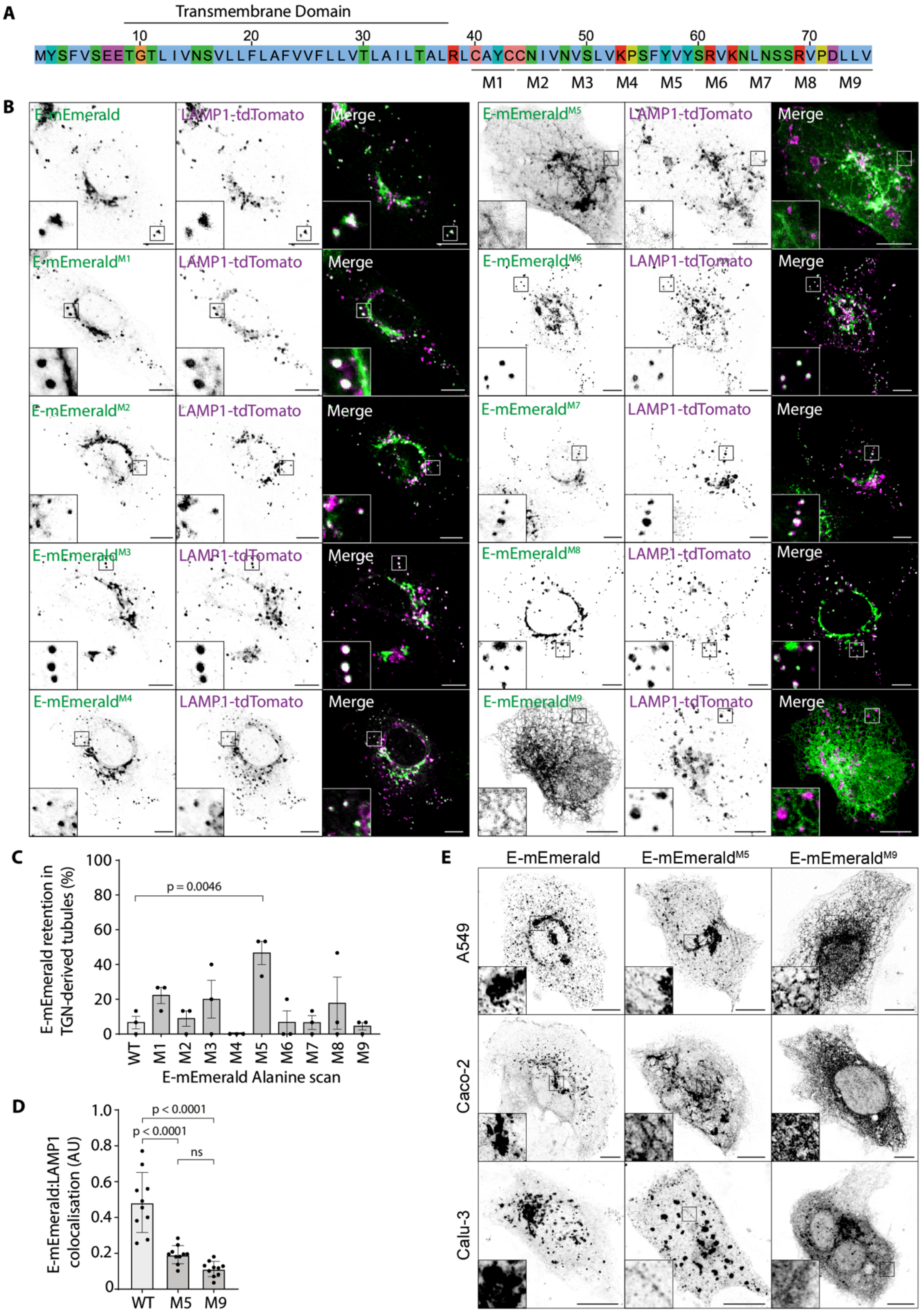

A
Transmembrane Domain
10
20
30
40
50
60
70
MYSFVSEETGTLIVNSVLLFLAFVVFLLVTLAILTALRLCAYCCNIVNVSLVKPSFYVYSRVKNLNSSRVPDLLV
M1
M2
M3
M4
M5
M6
M7
M8
M9
B
E-mEmerald
LAMP1-tdTomato
Merge
E-mEmerald^M1
E-mEmerald^M2
E-mEmerald^M3
E-mEmerald^M4
E-mEmerald^M5
E-mEmerald^M6
E-mEmerald^M7
E-mEmerald^M8
E-mEmerald^M9
C
E-mEmerald retention in TGN-derived tubules (%)
p = 0.0046
WT
M1
M2
M3
M4
M5
M6
M7
M8
M9
E-mEmerald Alanine scan
D
E-mEmerald:LAMP1 colocalisation (AU)
p < 0.0001
p < 0.0001
ns
WT
M5
M9
E
E-mEmerald
E-mEmerald^M5
E-mEmerald^M9
A549
Caco-2
Calu-3

Fig. S2. Alanine-scanning mutagenesis of the cytoplasmic tail of E. (**A**) Schematic of alanine-scanning mutagenesis of the C-terminus of E. In mutants M1 – M9, this indicated amino acids were exchanged for Alanine. (**B**) Representative images of VeroE6 cells expressing the indicated versions of E-mEmerald and LAMP1-tdTomato from at least 15 imaged cells per condition. (**C**) Quantification of E-mEmerald retention of in Golgi-derived tubules in VeroE6 cells transfected with the indicated Alanine-scanning mutants from Fig. S2B. Quantification is from at least 15 imaged cells per experiment from N = 3 independent experiments. Mean ± S.E. is displayed, with statistical significance determined by one-way ANOVA with Dunnett's correction. (**D**) Mander's colocalization co-efficient of E-mEmerald expressing VeroE6 cells stained with antisera raised against LAMP1. Mean ± S.D. of E-mEmerald signal in LAMP1 positive lysosomes provided from 10 imaged cells. Significance calculated by 1-way ANOVA with Tukey's correction. (**E**) A549, Caco-2 or Calu-3 cells were transfected with plasmids encoding the indicated E-mEmerald, E-mEmerald^{M5} or E-mEmerald^{M9} images representative of between 39 and 60 imaged cells (A549); 31 and 38 imaged cells (Caco-2); 7 and 11 imaged cells (Calu-3). In microscopy panels, scale bars are 10 μm.

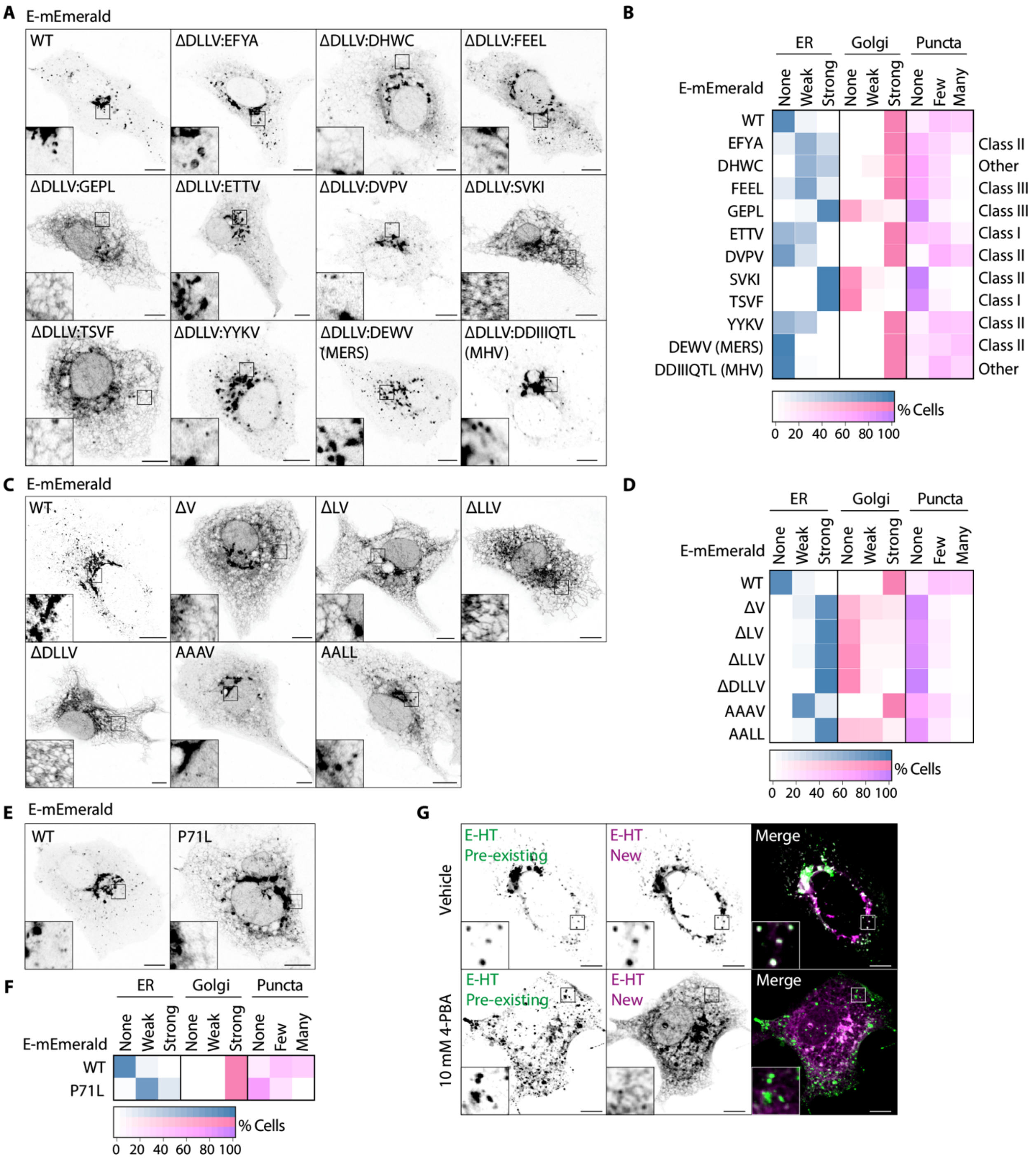

Fig. S3. The C-terminus of coronaviral E proteins encodes an ER-export motif (A, B) Representative images and quilt quantification from 50 imaged VeroE6 cells per condition transfected with a panel of E-mEmerald mutants in which the putative C-terminal PDZ-ligand in E (DLLV) was replaced by PDZ-ligands of different classes, or with chimaeric sequences from Middle East Respiratory Virus (MERS)-CoV or Murine Hepatitis Virus (MHV)-CoV (Strain S). The class of PDZ ligand is indicated, with Class I defined by -X-[S/T]-X-ϕ, Class II defined by -X-ϕ-X-ϕ and Class III defined by -X-[D/E/K/R]-X-ϕ (*54*) (**C, D**) Representative images and quilt quantification from 50 imaged VeroE6 cells per condition transfected with a panel of E-mEmerald mutants in which residues in the C-terminal ER-export motif (DLLV) were sequentially deleted or were replaced by C-terminal hydrophobic (AAAV) or di-hydrophobic (AALL) sequences. (**E, F**) Representative images of VeroE6

cells transfected with E-mEmerald or E-mEmerald^{P71L} with subcellular localization quantified (F) from 50 imaged cells. (**G**) Representative images of VeroE6 cells transfected with E-HT and illuminated with Oregon Green HaloTag ligand (green), released into dye-free media in the presence or absence of 4-PBA for 6 hours and then re-stained with TMR HaloTag ligand to illuminate newly synthesised E-HT (magenta). Images representative of 50 imaged cells per experiment across 3 independent experiments. The newly synthesized E-HT images from this panel are additionally presented in Fig. 2J. In microscopy panels, scale bars are 10 μm.

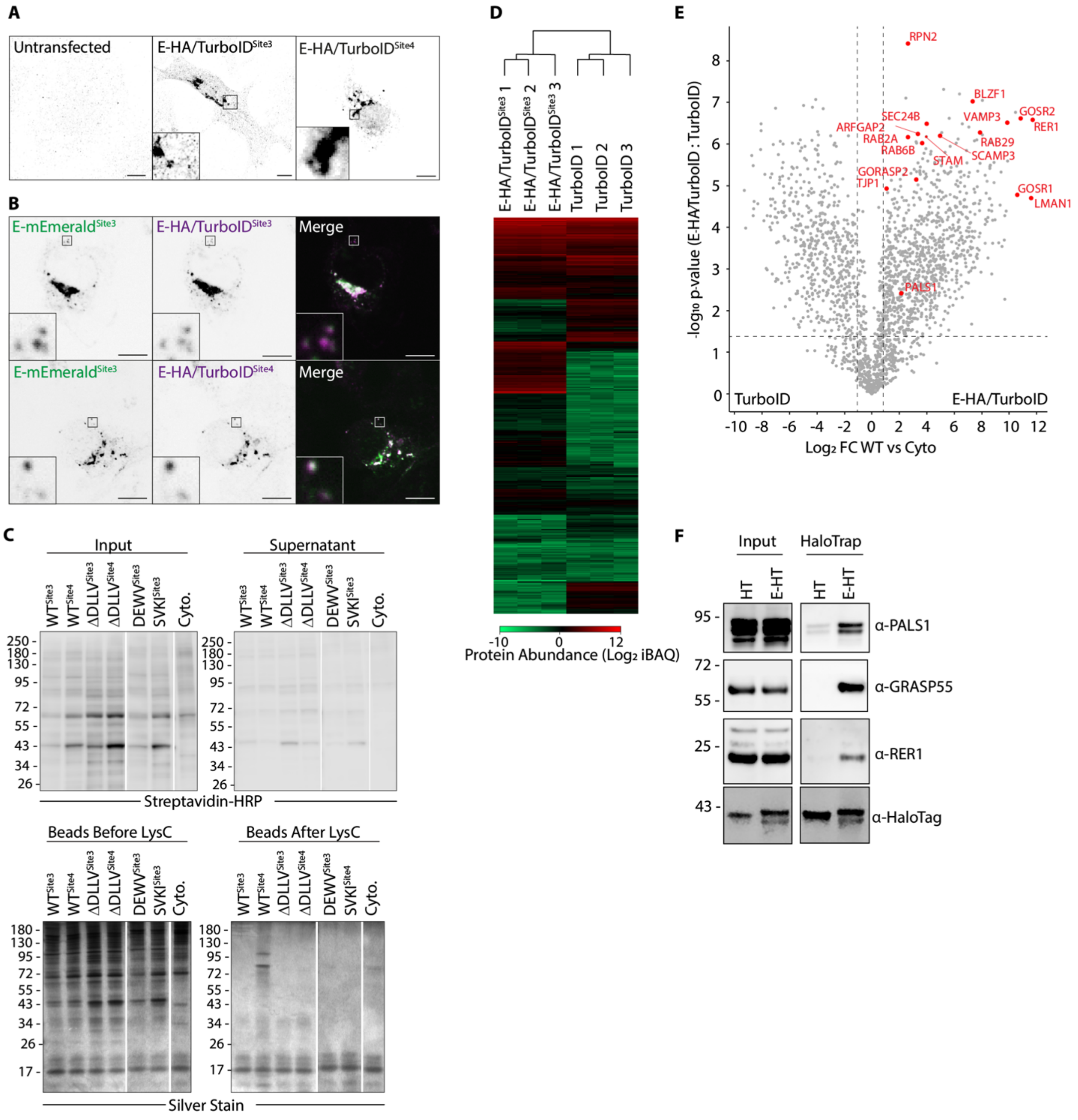

Fig. S4. Validation of TurboID-tagging of E proteins. (**A**) VeroE6 cells transfected with plasmids encoding E-HA/TurboID were fixed and stained with antisera raised against HA. Images representative of 15 or 6 imaged cells. (**B**) 293T cells transfected with plasmids encoding E-HA/TurboID and E-mEmerald^{Site3} were fixed and stained with antisera raised against HA. Pearson's colocalisation coefficient (E-mEmerald^{Site3} vs E-HA/TurboID^{Site3}, 0.915 ± 0.038; E-mEmerald^{Site3} vs E-HA/TurboID^{Site4}, 0.883 ± 0.054) calculated from 8 imaged cells per condition, mean ± S.D. (**C**) Quality control for TurboID-biotinylation-neutravidin pulldown and protein cleavage from beads. Samples (E-HA/TurboID^{Site3}, E-HA/TurboID^{Site4}, E$^{\Delta DLLV}$-HA/TurboID^{Site3}, E$^{\Delta DLLV}$-HA/TurboID^{Site4}, E$^{\Delta DLLV+DEWV}$-HA/TurboID^{Site3}, E$^{\Delta DLLV+SVKI}$-HA/TurboID^{Site3}, or HA/TurboID (cyto)) were extracted from the post-TurboID reaction pulldown input (Input) and supernatant after neutravidin bead incubation (Supernatant), resolved using SDS-PAGE, and blotted using Streptavidin-HRP. Proteins captured on neutravidin beads before and after LysC cleavage were obtained by boiling neutravidin beads in Laemmli buffer with β-mercaptoethanol, were resolved using SDS-PAGE and detected by Silver Stain. Conditions E-

HA/TurboID^{Site4}, $E^{\Delta DLLV}$-HA/TurboID^{Site3}, $E^{\Delta DLLV}$-HA/TurboID^{Site4}, $E^{\Delta DLLV+DEWV}$-HA/TurboID^{Site3} and $E^{\Delta DLLV+SVKI}$-HA/TurboID^{Site3} are discussed in Fig. S5. (**D**) Hierarchical clustering performed on the median adjusted IBAQ values of each proteomic sample calculated and plotted by average Euclidean distance. Colour scale indicates protein abundance as measured by log_2 transformed iBAQ values. (**E**) Volcano plot depicting proteins recovered from neutravidin pulldown from 293T cells expressing TurboID or E-HA/TurboID fusions and subject to a 20-minute biotinylation prior to lysis. Volcano plot constructed from N = 3 biological repeats. A selection of proteins strongly enriched by E-HA/TurboID is displayed in red. Percentages were counted from proteins that changed abundance by more than 2-fold and were statistically significant at $p < 0.05$, as determined by FDR-corrected two-tailed T-tests. (**F**) Cell lysates and HaloTrap-captured fractions from 293T cells transfected with the indicated E-HT^{Site3} fusions were resolved by SDS-PAGE and examined by western blotting with the indicated antisera (N = 3).

A

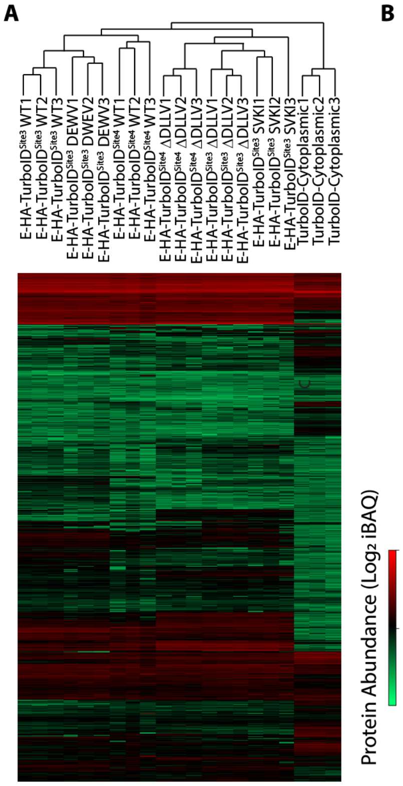

B

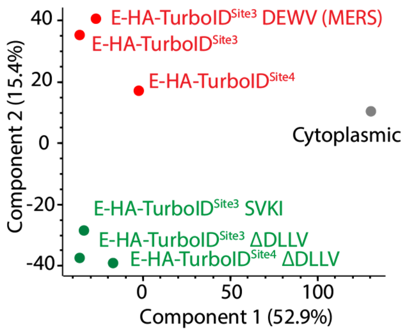

C

PDZ Proteins	FC	p-value
TJP1	5.79	5.35
TJP2	5.74	4.59
PARD3	2.34	4.24
AHNAK	3.27	3.88
GORASP2	1.90	3.39
MLLT4	2.36	3.47
PTPN13	4.48	3.24
MPP6	2.87	3.08
CNKSR3	4.20	3.02
ARHGAP21	2.34	2.74
MPP5	3.86	2.93
GOPC	1.15	2.49
SCRIB	2.91	2.57
SNTB2	2.06	2.01
DLG1	3.48	1.98
MAGI1	1.90	1.80

D

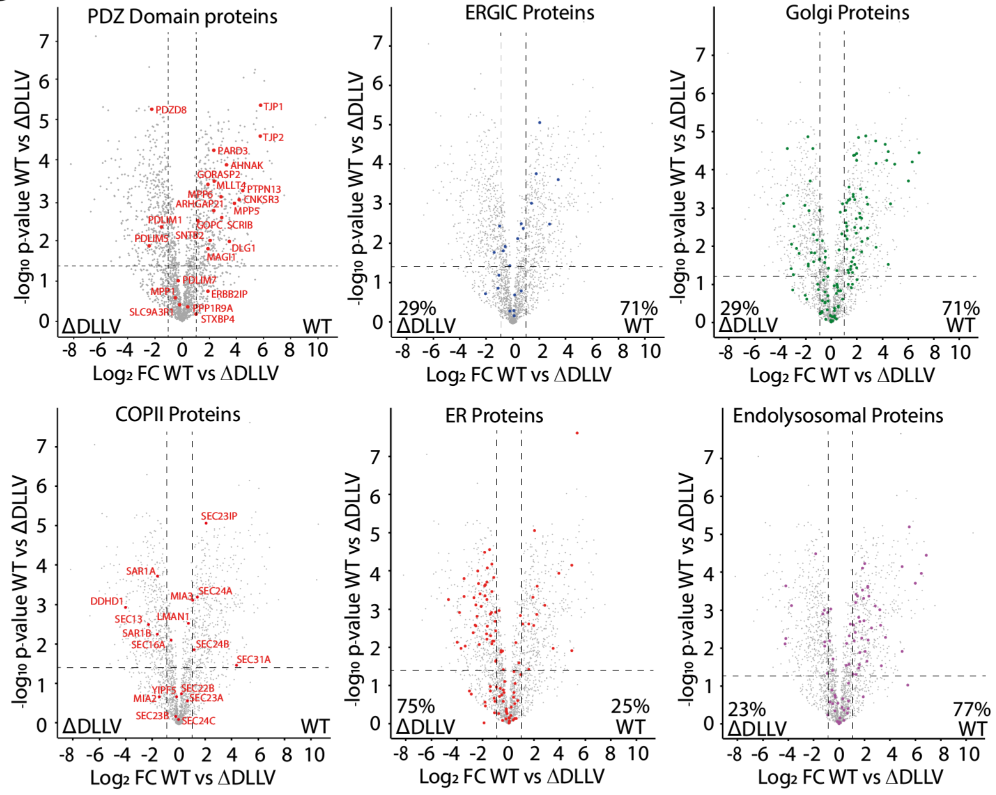

Fig. S5. Comparative proximity biotinylation of ER-export proficient and ER-export defective versions of E. (**A**) Hierarchical clustering performed on the median adjusted IBAQ values of each proteomic sample calculated and plotted by average Euclidean distance. Colour scale indicates protein abundance as measured by log_2 transformed iBAQ values. (**B**) Principal component analysis (PCA) on median averaged data across the three repeats for each sample. Red dots/text indicate WT-like E proteins, green dots/text indicates ΔDLLV-like E proteins, and grey indicates the cytoplasmic control. (**C**) Tabular depiction of PDZ-domain containing proteins recovered from comparative proteomics of WT and ΔDLLV versions of E-HA/TurboID. (**D**) Volcano plots depicting proteins recovered from a neutravidin pull down from 293T cells expressing either E-HA/TurboID^{Site3} or E-HA/TurboID^{Site3} ΔDLLV, and subject to a 20-minute biotinylation. N = 3. Gene Ontology was used to assign recovered proteins to subcellular localisations. PDZ-domain containing proteins were annotated on the volcano plot. Percentages were counted from proteins that changed abundance by more than 2-fold and were statistically significant at $p < 0.05$, as determined by FDR-corrected two-tailed T-tests.

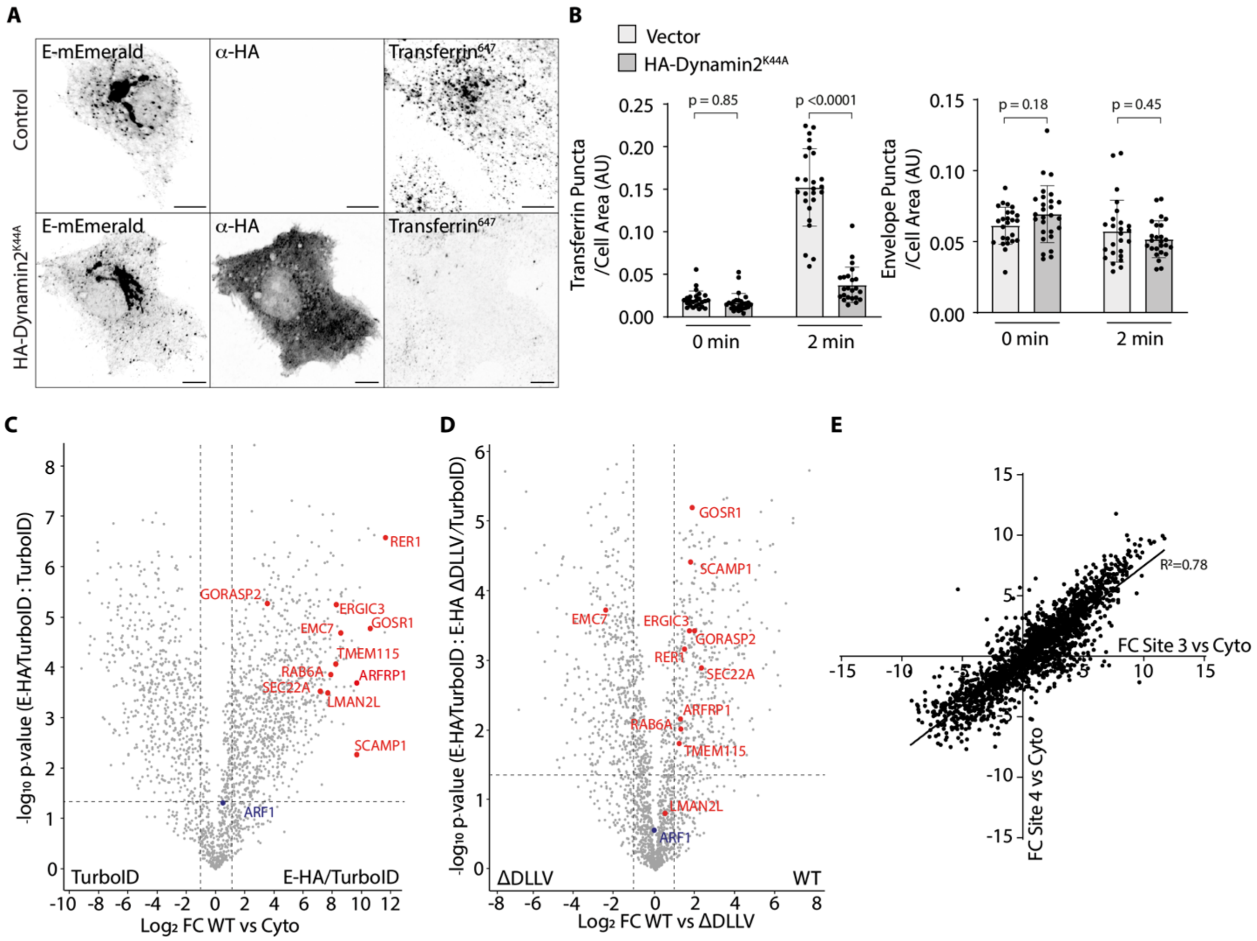

Fig. S6. Investigation of Golgi-to-lysosome trafficking of SARS-CoV-2 E. (A). Representative images of VeroE6 cells transfected for 18 hours with plasmids encoding E-mEmerald and either an empty vector or HA-Dynamin2^{K44A} and then incubated with Alexa^{647}-labelled Transferrin for 2 minutes. (**B**) Quantification of Transferrin puncta in cells from A, mean ± S.D. displayed, statistical significance determined by 2-tailed T-Test from 27 imaged cells per condition. Quantification of E-mEmerald puncta in cells from A. Mean ± S.D. displayed with statistical significance determined by 2-tailed T-Test from 25 imaged cells. (**C, D**) Volcano plot depicting proteins recovered from a neutravidin pulldown from 293T cells expressing HA/TurboID (cyto) or E-HA/TurboID (WT) fusions and subject to a 20-minute biotinylation prior to lysis. Volcano plot constructed from N = 3 biological repeats and normalized against cytoplasmic TurboID (C) or E-HA/TurboID^{DDLLV} (D). Percentages were counted from proteins that changed abundance by more than 2-fold and were statistically significant at $p < 0.05$, as determined by FDR-corrected two-tailed T-tests. The position of 12 significantly enriched membrane trafficking proteins (fold change > 6 and -log$_{10}$ based p-values of > 3; GORASP2 and SCAMP1 were additionally selected due to their extreme placement in terms of significance or fold change) selected for our knockout screen are depicted in red. The ARFRP1-related GTPase, ARF1, is highlighted in blue and was not significantly enriched. (**E**) Correlation plot of candidates returned by E-HA/TurboID^{Site3} vs E-HA/TurboID^{Site4}. In microscopy panels, scale bars are 10 μm.

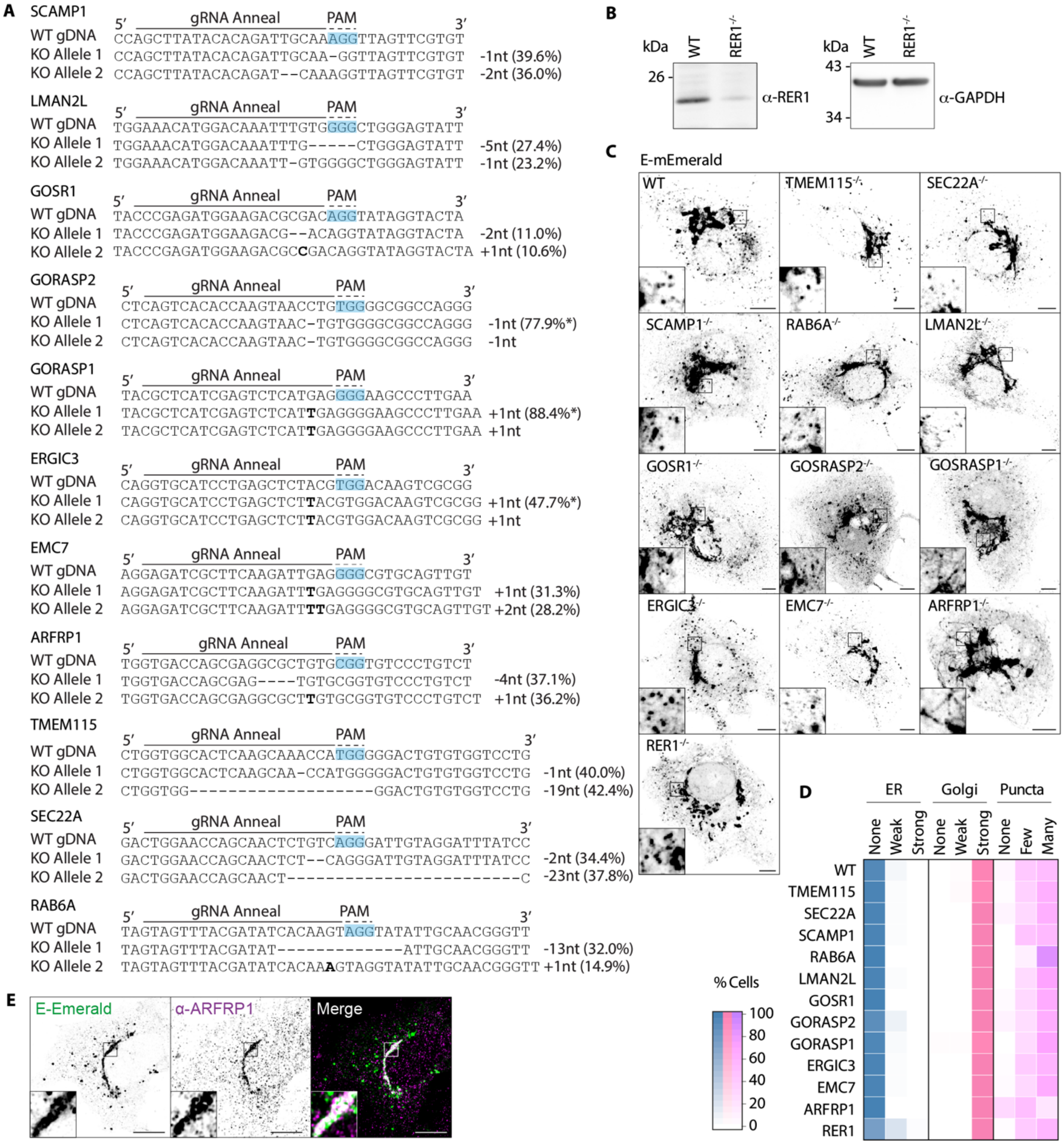

Fig. S7. Examination of E-mEmerald trafficking in a panel of knockout VeroE6 cells. (**A**) Genomic locus of the gRNA-annealing region and protospacer adjacent motif (PAM) of each target, and Next Generation Sequencing of allele-specific indels in edited VeroE6 clones, including allele percentages of the two most common reads. (**B**) Resolved lysates from WT or RER1$^{-/-}$ VeroE6 cells were examined by western blotting with antisera raised against RER1 or GAPDH. (**C, D**) Representative images and quantification of subcellular distribution of the indicated knockout VeroE6 cells transfected with a plasmid encoding E-mEmerald. Quantification performed from 50 cells per condition. (**E**) VeroE6 cells expressing E-mEmerald and stained against ARFRP1 and the amount of E-mEmerald in ARFRP1-positive membranes was calculated by Mander's colocalisation coefficient (0.379 ± 0.106, mean ± S.D. from 26 imaged cells). In microscopy panels, scale bars are 10 μm.

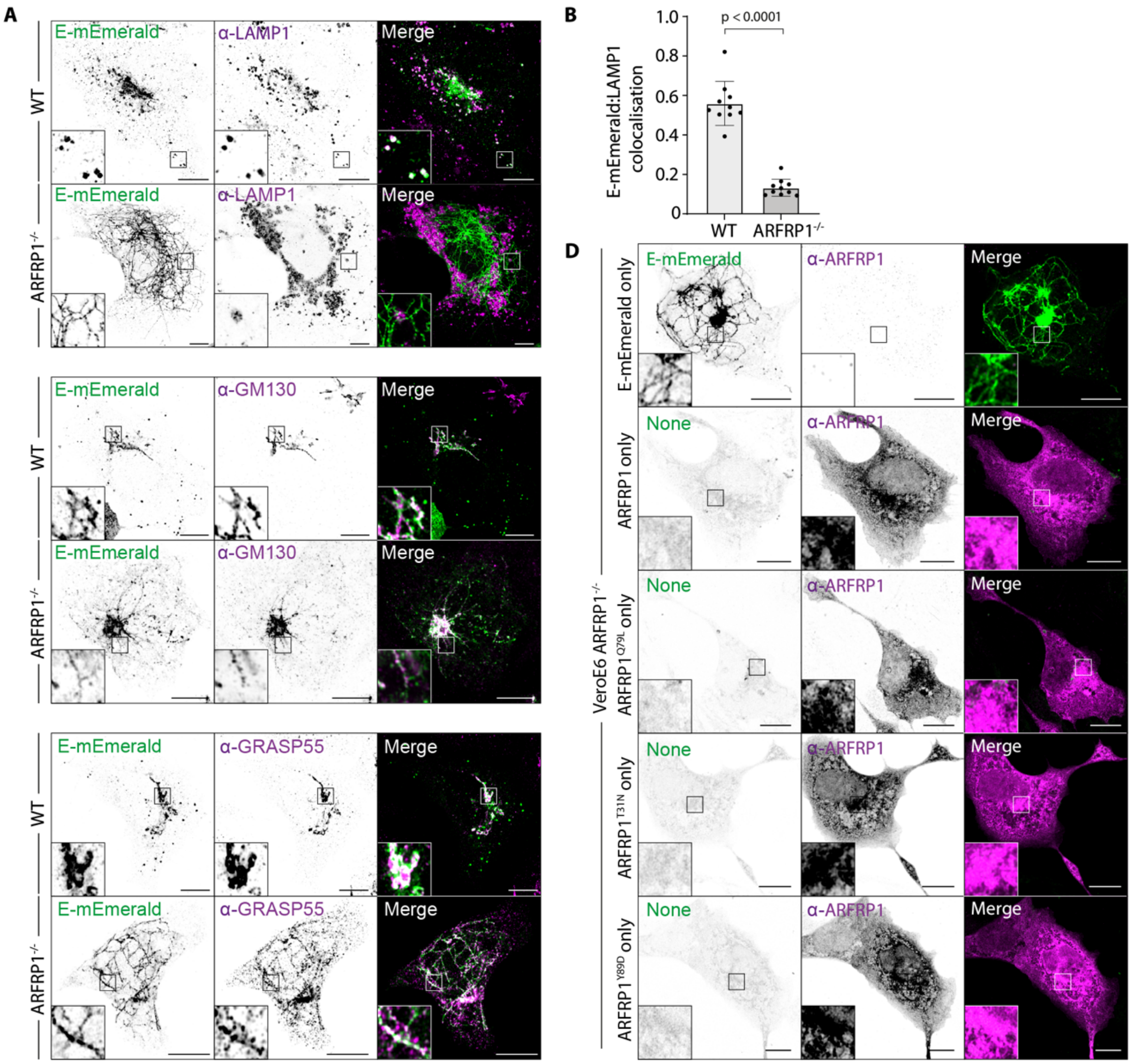

C

```
ARFRP1 MYTLLSGLYKYMFQKDEYCILILGLDNAGKTTFLEQSKTRFNKNYKGMSLSKITTTVGLNIGTVDVGKARLMFWDLGGQEELQSL  85
ARF1   MGNIFANLFKGLFGKKEMRILMVGLDAAGKTTILYKLKLG--------EIVTTIPTIGFNVETVEYKNISFTVWDVGGQDKIRPL  77
       * .::::.*:* :* *.*  **::*** *****:* : *         .: .   *:*:*: **:  :  : .**:***:::: *

ARFRP1 WDKYYAECHGVIYVIDSTDEERLAESKQAFEKVVTSEALCGVPVLVLANKQDVETCLSIPDIKTAFSDCTSKIGRRDCLTQACSA  170
ARF1   WRHYFQNTQGLIFVVDSNDRERVNEAREELMRMLAEDELRDAVLLVFANKQDLPNAMNAAEITDKLGL--HSLRHRNWYIQATCA  160
       * :*: : :*:*:*:**.*.**: *::: : ::::.: * .. :**:*****: ..:.  :*.  :.    .: :*:   ** .*

ARFRP1 LTGKGVREGIEWMVKCVVRNVHRPPRQRDIT  201
ARF1   TSGDGLYEGLDWLSNQLRNQK----------  181
       :*.*: **::*: : : .:
```

Fig. S8. E-mEmerald is retained in Golgi-derived tubules in the absence of ARFRP1. (**A**) WT or ARFRP1$^{-/-}$ VeroE6 cells were transfected with E-mEmerald, fixed and stained with antisera raised against LAMP1, GM130 or GRASP55. Images representative of between 7 and 26 imaged cells per condition. (**B**) Mander's coefficient, mean ± S.D. of E-mEmerald signal in LAMP1 positive lysosomes provided from 10 imaged cells. Significance calculated by an unpaired 2-tailed T-test. (**C**) Clustal Omega alignment of ARF1 and ARFRP1 with residues involved in GTPase activity (ARF1: Q71, T31; ARFRP1: Q79, T31) in red and residues involved in the hydrophobic effector patch (ARF1 Y81; ARFRP1 Y89) in green. (**D**) Representative images of ARFRP1$^{-/-}$ VeroE6 cells with the single plasmid transfections as indicated and stained with antisera against ARFRP1. Images representative of between 10 and 15 imaged cells per condition. In microscopy panels, scale bars are 10 μm.

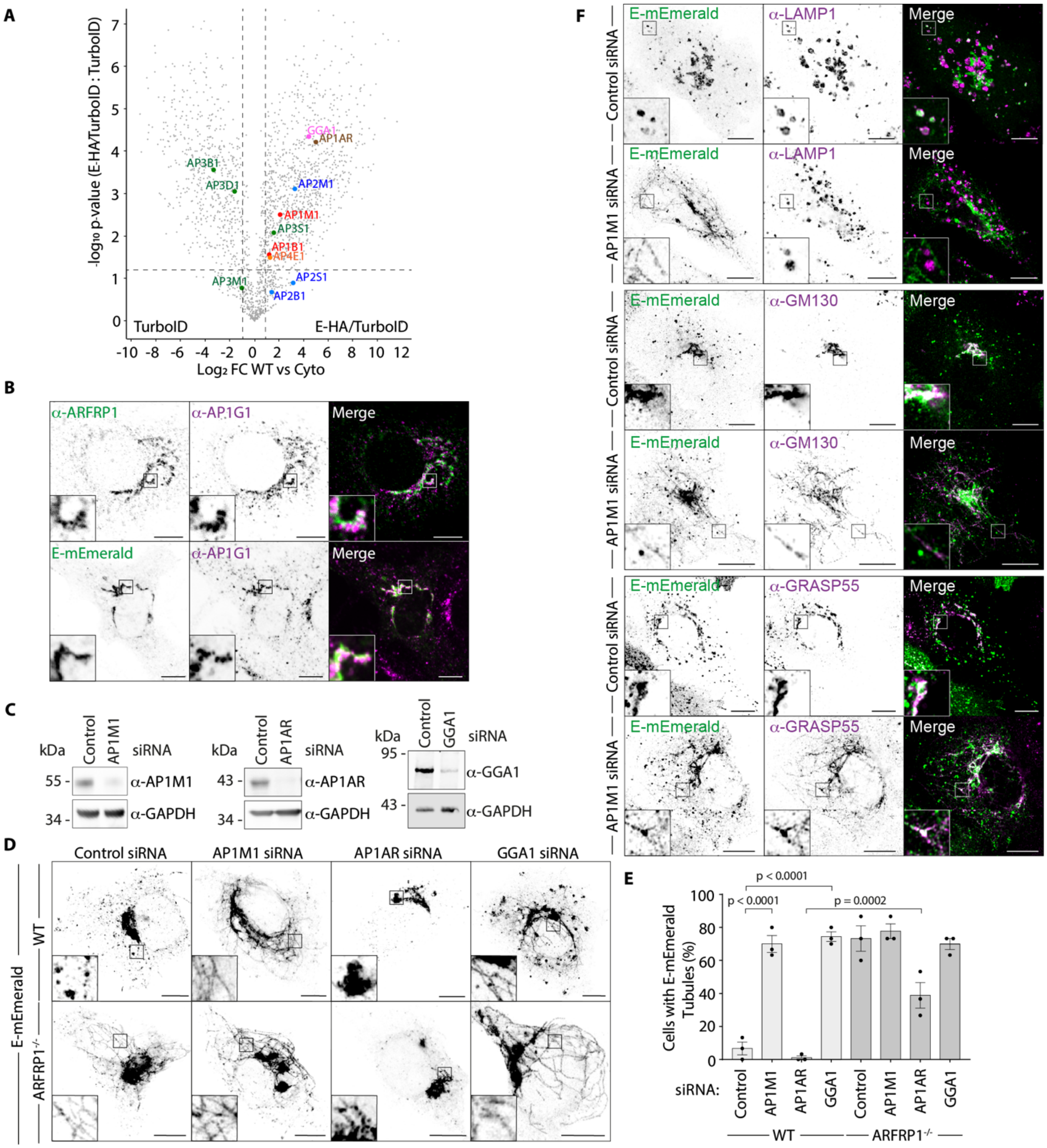

Fig. S9. The role of ARFRP1 in coordinating AP-1 at the Golgi for Golgi-to-lysosome trafficking of E. (A) Volcano plot depicting proteins recovered from neutravidin pull down from 293T cells expressing either HA-TurboID or E-HA/TurboID and subject to a 20-minute biotinylation. N = 3. All adaptor proteins (AP) identified are highlighted, with colours corresponding to the different AP complexes. Percentages were counted from proteins that changed abundance by more than 2-fold and were statistically significant at $p < 0.05$, as determined by FDR-corrected two-tailed T-tests. (**B**) VeroE6 cells, or VeroE6 cells transfected with E-mEmerald, were fixed and stained with antisera raised against AP1G1 or ARFRP1 as indicated. Images representative of 12 or 14

imaged cells respectively. (**C**) Resolved cell lysates from VeroE6 cells that had transfected with control siRNA or siRNA targeting AP1M1, AP1AR or GGA1 were examined by western blotting with antisera raised against AP1M1, AP1AR, GGA1 or GAPDH. (**D, E**) Representative images of WT or ARFRP1$^{-/-}$ VeroE6 cells transfected with the indicated siRNA and a plasmid encoding E-mEmerald (D) with quantification of the number of cells displaying E-mEmerald tubules reported (E). 15 cells per experiment were scored from N = 3 independent experiments. Significance calculated by one-way ANOVA comparing WT control against WT AP1M1 siRNA, WT control against GGA1 siRNA, and WT AP1AR siRNA against ARFRP1$^{-/-}$ AP1AR siRNA, with Šidák's correction for multiple testing. (**F**) A549 cells were transfected with control siRNA or siRNA targeting AP1M1 and a plasmid encoding E-mEmerald. Cells were fixed and stained with antisera raised against LAMP1, GM130 or GRASP55 as indicated. Images representative of between 5 and 14 imaged cells per condition. In microscopy panels, scale bars are 10 µm.

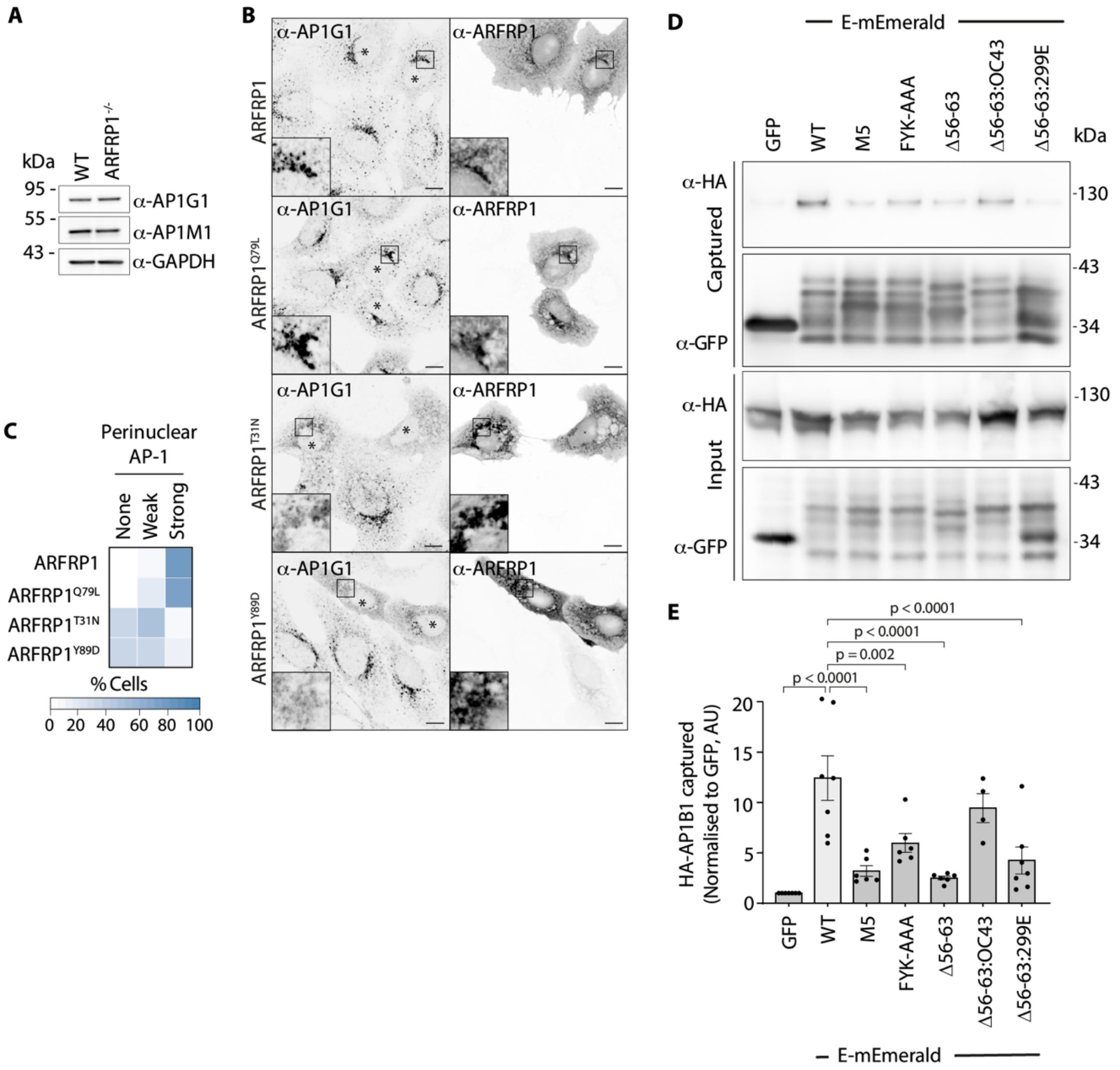

Fig. S10. The β-coronavirus specific insertion binds AP-1. (**A**) Resolved cell lysates from WT or ARFRP1$^{-/-}$ VeroE6 cells were examined by western blotting with antisera raised against GAPDH, AP1G1 or AP1M1. (**B**, **C**) VeroE6 cells were transfected with plasmids encoding the indicated ARFRP1 proteins and stained with antisera raised against ARFRP1 or AP1G1 (B). Transfected cells indicated by asterisks. Perinuclear localisation of AP1G1 was scored in the accompanying quilt (C) from 50 imaged cells per condition. Acquisition settings were optimised for overexpressed ARFRP1 staining. (**D**) Cell lysates and GFP-Trap immunoprecipitations of 293T cells co-transfected with plasmids encoding GFP or the indicated E-mEmerald constructs and HA-AP1B1 were resolved by SDS-PAGE and examined by western blotting with antisera raised against HA or GFP. (**E**) Quantification of data from D from N = 4 to 7 independent experiments as indicted, significance calculated by 1-way ANOVA with Dunnet's correction. In microscopy panels, scale bars are 10 μm.

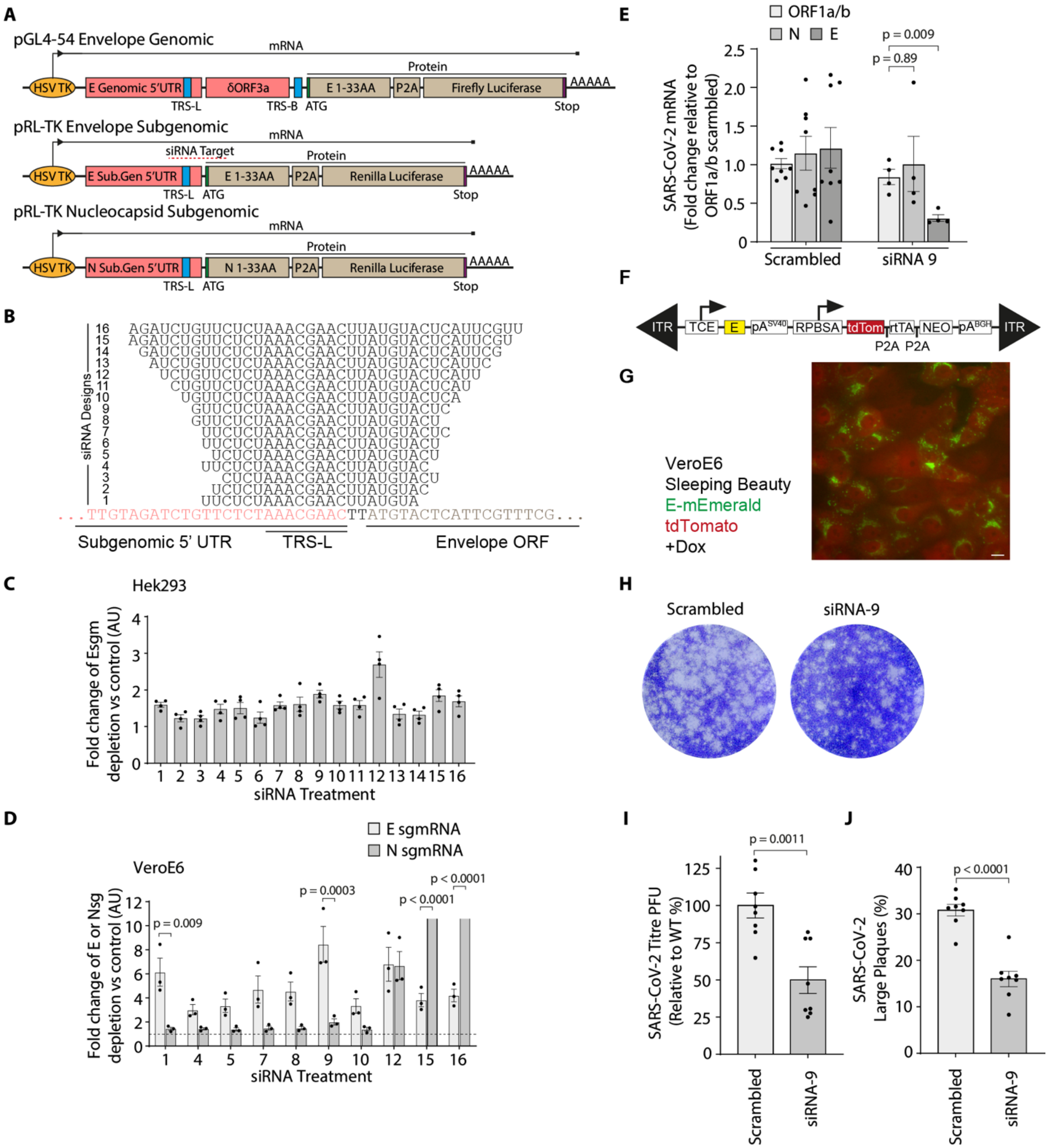

Fig. S11. Validation of siRNA designed to target SARS-CoV-2 Envelope sgmRNA. (**A**) Schematic showing the design of a dual-luciferase assay reporter to test siRNA efficacy and specificity against E subgenomic mRNA (sgmRNA). TRS elements and the annealing region of the E-sgmRNAs are labelled. (**B**) Schematic showing the design of E-sgmRNA targeting siRNAs relative to E-sgmRNA. (**C**) For initial screening, Hek293 cells were transfected with 20 µM of control or E-sgmRNA targeting siRNAs and after 24 hours were transfected with a 1:1 ratio of E-subgenomic/E-genomic dual-luciferase assay reporter plasmids. After 24 hours, luminescence of Firefly and Renilla luciferases was assessed. The ratio of E-sgmRNA reporter and E-genomic reporter was calculated and a fold change relative to scrambled siRNA plotted. Mean ± S.E. is displayed, with N = 3. Relative depletion of E-sgmRNA reported as >1. (**D**) Promising siRNAs from Hek293 cells (C) were tested for efficiency

and specificity in VeroE6 cells. VeroE6 were transfected with siRNAs and either the E-subgenomic/E-genomic dual luciferase reporter, or N-subgenomic/E-genomic dual luciferase reporter, as above. Dual luminescence was recorded as described above. Data plotted shows the fold change of the ratio between E-sgmRNA reporter or N-sgmRNA reporter and E-genomic reporter for E-sgmRNA siRNA treatments, relative to a scrambled control siRNA. Mean ± S.E. is displayed, with N = 3, with statistical testing using a 2-way ANOVA using the Šidák correction. Relative depletion of E-sgmRNA or N-sgmRNA reported as > 1. Potent depletion of N-sgmRNA was obtained with siRNA 15 and 16, values for which (15 = 47.54 ; 16 = 34.86) are omitted from the plot. (**E**) VeroE6 cells were treated with 10 pmol E-sgmRNA targeting siRNA-9, or scrambled siRNA for 20 hours before being infected with SARS-CoV-2 (hCoV-19/England/02/2020) at 1 PFU/cell for 2 hours. After 24 hours, reverse transcription was performed and cDNA levels of SARS-CoV-2 ORF1ab, E and N sgmRNAs were quantified. The data plotted shows viral mRNA expression normalised to actin plotted relative to normalised ORF1a/b expression with the scrambled siRNA treatment. Mean ± S.E. is displayed, with N equal to the number of data points displayed. (**F**) Cartoon of pSBTet-RN. (**G**) VeroE6 cells constitutively expressing tdTomato and expressing a doxycycline-inducible E-mEmerald from the Sleeping Beauty retrotransposition vector were treated with doxycycline and imaged. Images representative of 5 imaged fields of view. (**H**) SARS-CoV-2 viruses from VeroE6 cells treated with either scrambled siRNA or E-sgmRNA targeting or siRNA-9 were generated. A diluted stock was applied to confluent VeroE6 cells for 30 minutes. Infected cells were grown for 3 days before being fixed and stained with 0.2% toluidine blue. Images show representative plaque formation. (**I**) Quantification of plaque formation represented as titre (PFU/ml) using the ViralPlaque FIJI macro. Mean ± S.E. presented from N = 8, with significance calculated by a 1-way ANOVA with Dunnet's correction comparing scrambled to E-sgmRNA siRNAs. (**J**) Quantification of the percentage of large plaques *vs* total plaques from plaque assays. Large plaques were defined by having an area greater than 0.82 mm^2 and measured using the ViralPlaque macro in FIJI. Mean ± S.E. presented from N = 8, with significance calculated by a 1-way ANOVA comparing scrambled to E-sgmRNA siRNAs with Dunnet's correction. In microscopy panels, scale bars are 10 μm.

Supplementary Data Tables

Data S1 (separate file). All data from label-free quantification of proximity biotinylation proteomics of HA-TurboID tagged SARS-CoV-2 E, SARS-CoV-2 E mutants, and HA-TurboID cytoplasmic controls.

Data S2 (separate file). Data subset of label free quantification data of proximity biotinylation proteomics of HA-TurboID tagged SARS-CoV-2 E compared to HA-TurboID cytoplasmic control.

Data S3 (separate file). Data subset of label free quantification data of proximity biotinylation proteomics of HA-TurboID tagged SARS-CoV-2 E compared to SARS-CoV-2 E ΔDLLV.

Data S4 (separate file). Comparative analysis of proximal proteome reported herein against previously published proteomes for SARS-CoV-2 E.

Data S5 (separate file). siRNAs screened for E-sgmRNA targeting.

Data S6 (separate file). PCR primers for the validation of CRISPR knockout clones by next-generation sequencing.

Figure 3A

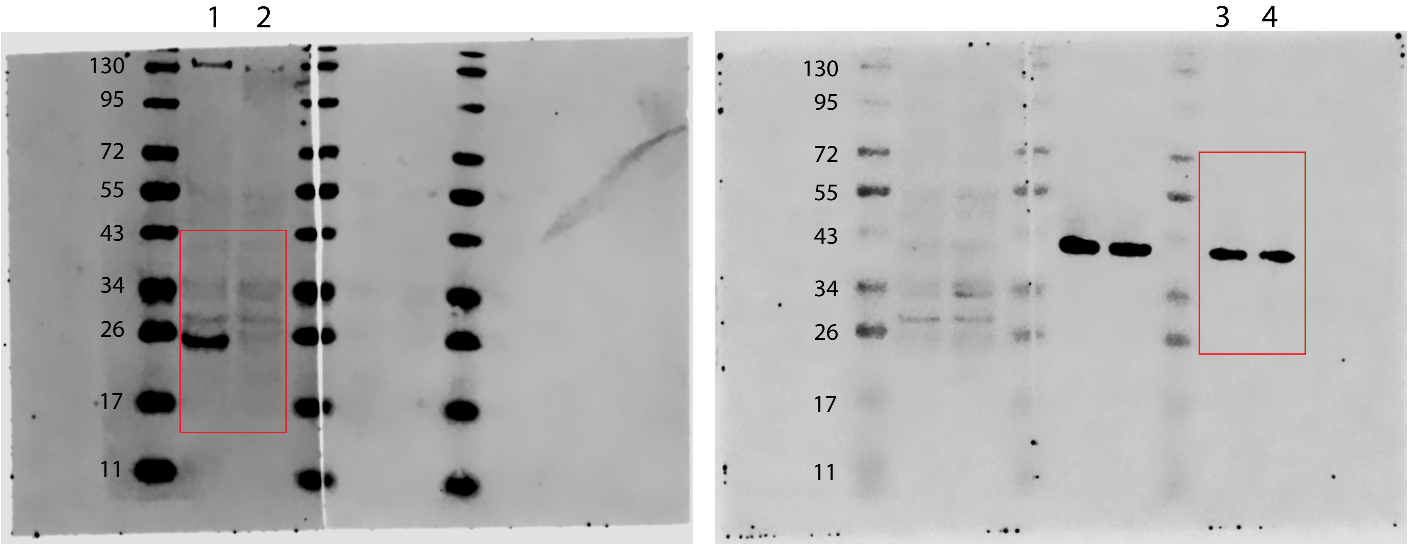

α-ARFRP1

α-GAPDH

Western Blot Lanes
1 - VeroE6 lysate
2 - VeroE6 ARFRP1 KO lysate
3 - VeroE6 lysate
4 - VeroE6 ARFRP1 KO lysate

Figure 5I

Low Exposure

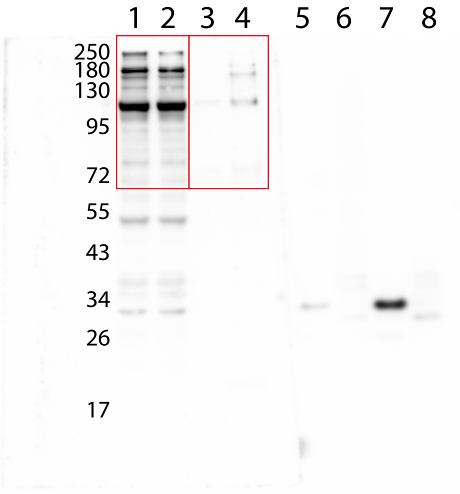

α-AP1B1 α-GFP

High Exposure

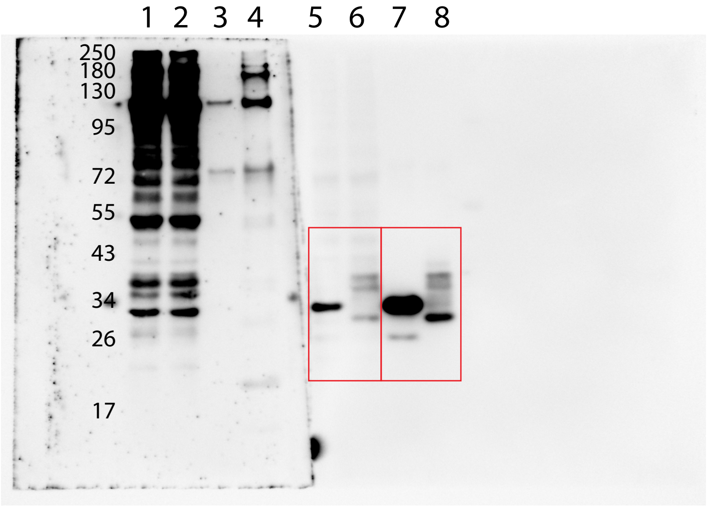

α-AP1B1 α-GFP

Western Blot Lanes
<u>Input</u>
1 - GFP
2 - E-Emerald
<u>Captured</u>
3 - GFP
4 - E-Emerald

<u>Input</u>
5 - GFP
6 - E-Emerald
<u>Captured</u>
7 - GFP
8 - E-Emerald

Figure 6E

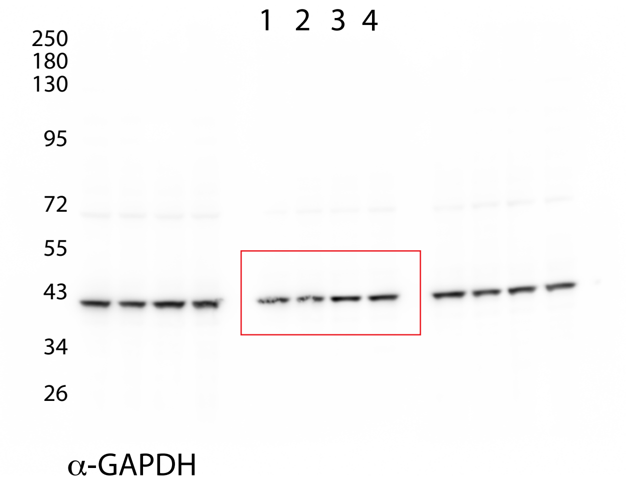

Western Blot Lanes

1 - E-WT + M/N/S
2 - E-N15AV25F + M/N/S
3 - E-M5 + M/N/S
4 - E-ΔDLLV + M/N/S

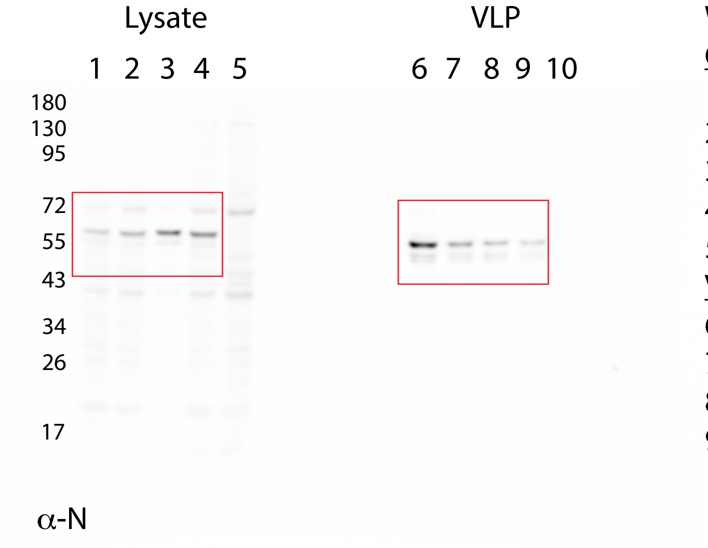

Western Blot Lanes
Cells
1 - E-WT + M/N/S
2 - E-N15AV25F + M/N/S
3 - E-M5 + M/N/S
4 - E-ΔDLLV + M/N/S
5 - Untransfected
VLPs
6 - E-WT + M/N/S
7 - E-N15AV25F + M/N/S
8 - E-M5 + M/N/S
9 - E-ΔDLLV + M/N/S
10 - Untransfected

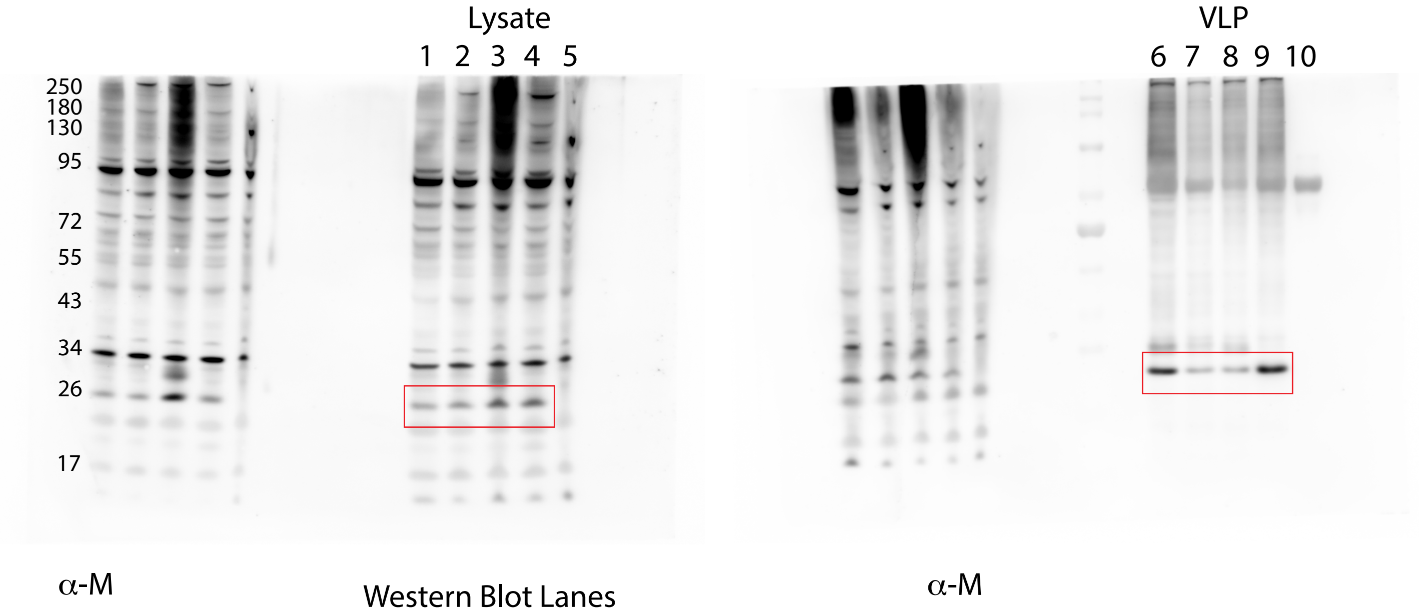

Western Blot Lanes
Lysate
1 - E-WT + M/N/S
2 - E-N15AV25F + M/N/S
3 - E-M5 + M/N/S
4 - E-ΔDLLV + M/N/S
5 - Untransfected

VLPs
6 - E-WT + M/N/S
7 - E-N15AV25F + M/N/S
8 - E-M5 + M/N/S
9 - E-ΔDLLV + M/N/S
10 - Untransfected

Figure S4C

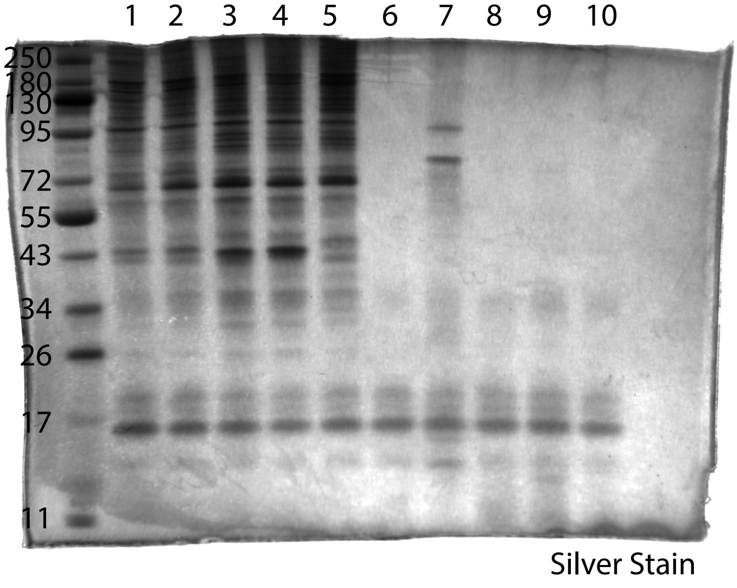

Silver Stain Lanes:

Beads Before LysC

1 - WT^{Site3}

2 - WT^{Site4}

3 - ΔDLLV^{Site3}

4 - ΔDLLV^{Site4}

5 - HA-TurboID at Envelope C-terminus

Beads After LysC

6 - WT^{Site3}

7 - WT^{Site4}

8 - ΔDLLV^{Site3}

9 - ΔDLLV^{Site4}

10 - HA-TurboID at Envelope C-terminus

Figure S4C

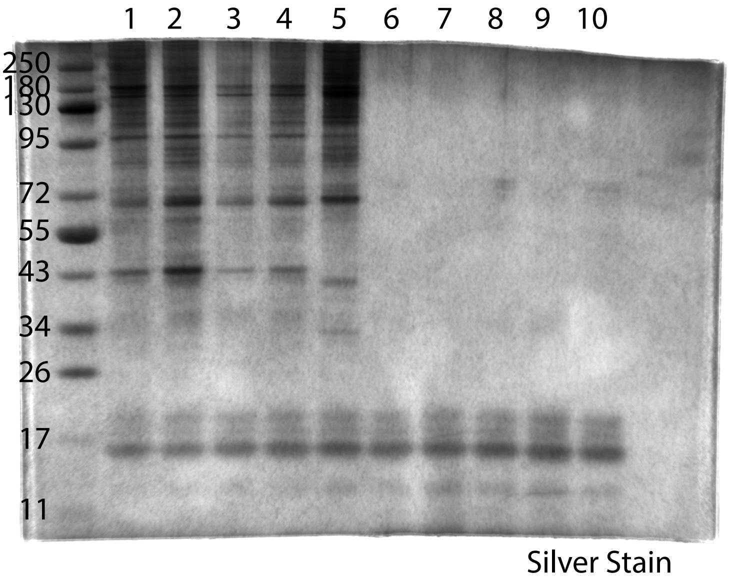

Silver Stain:

Beads Before LysC

1 - DLLV replaced by DEWV^{Site3}

2 - DLLV replaced by SVKI^{Site3}

3 - R61A K63A^{Site3}

4 - R61A K63A^{Site4}

5 - Emerald-TurboID

Beads After LysC

6 - DLLV replaced by DEWV^{Site3}

7 - DLLV replaced by SVKI^{Site3}

8 - R61A K63A^{Site3}

9 - R61A K63A^{Site4}

10 - Emerald-TurboID

Figure S4C

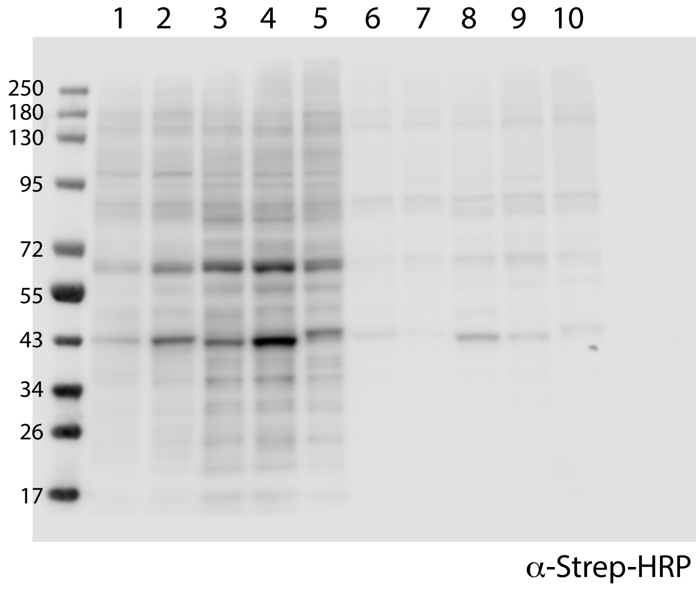

Strep-HRP Antibody Lanes:

Input

1 - WT^{Site3}

2 - WT^{Site4}

3 - ΔDLLV^{Site3}

4 - ΔDLLV^{Site4}

5 - HA-TurboID at Envelope C-terminus

Supernatant

6 - WT^{Site3}

7 - WT^{Site4}

8 - ΔDLLV^{Site3}

9 - ΔDLLV^{Site4}

10 - HA-TurboID at Envelope C-terminus

Figure S4C

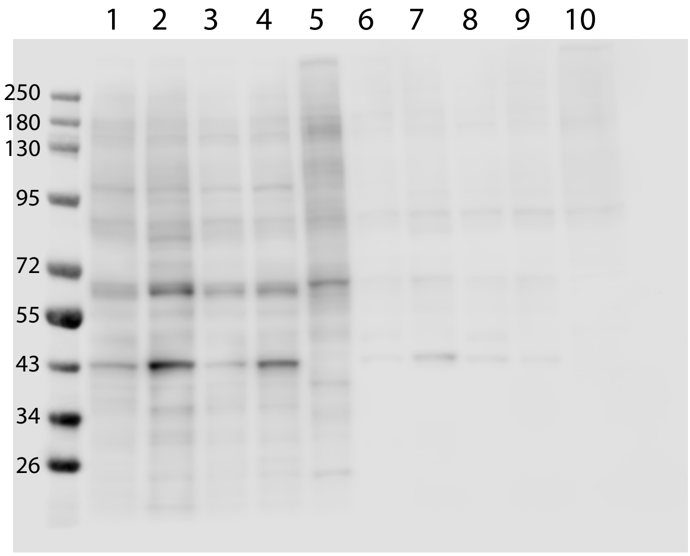

<u>Input</u>

1 - DLLV replaced by DEWV^{Site3}

2 - DLLV replaced by SVKI^{Site3}

3 - R61A K63A^{Site3}

4 - R61A K63A^{Site4}

5 - Emerald-TurboID

<u>Beads After LysC</u>

6 - DLLV replaced by DEWV^{Site3}

7 - DLLV replaced by SVKI^{Site3}

8 - R61A K63A^{Site3}

9 - R61A K63A^{Site4}

10 - Emerald-TurboID

α-Strep HRP

Figure S4F - RER1, PALS1

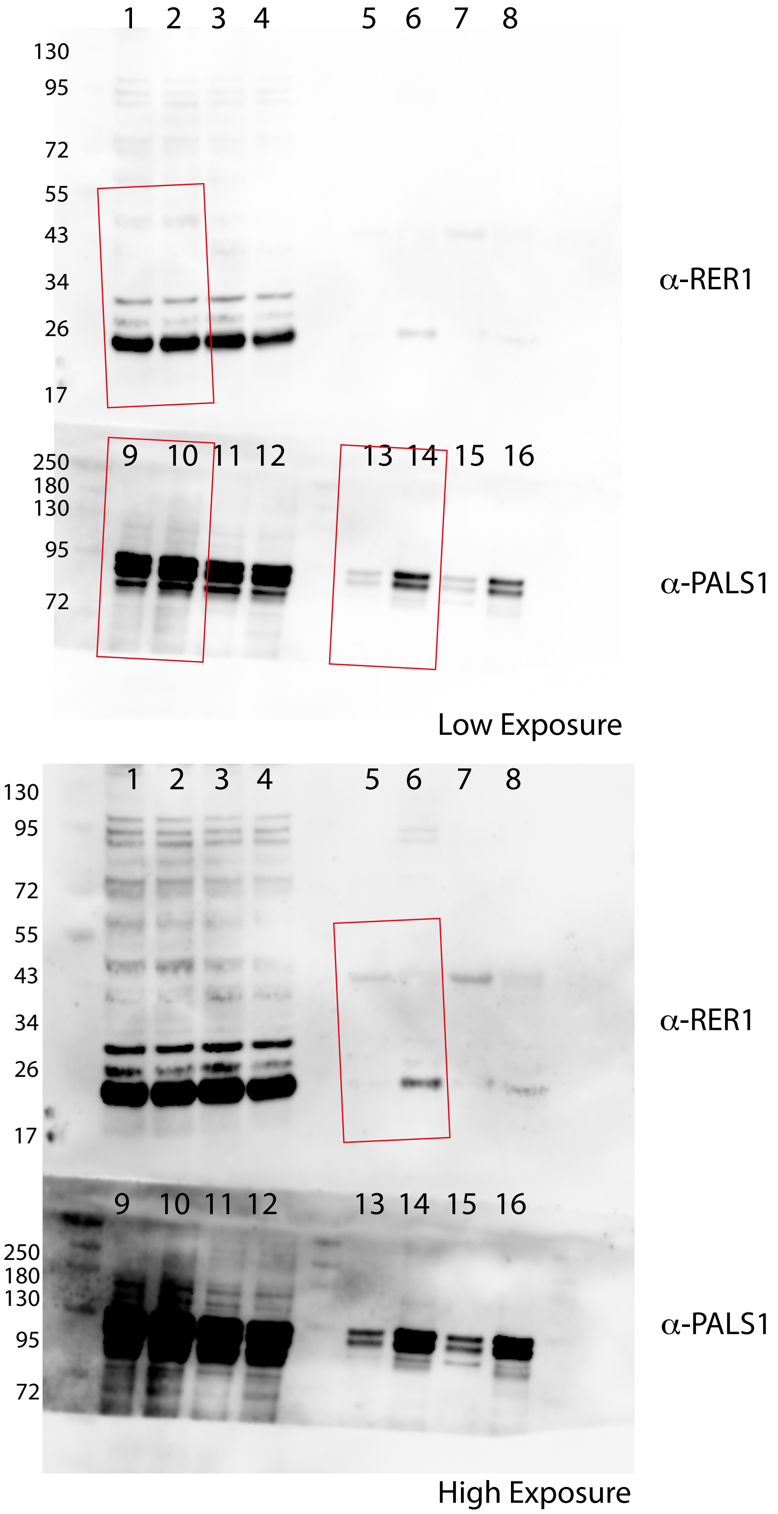

Western Blot Lanes

Input

1 - HT
2 - E-HT
3 - HT
4 - E-HT

Captured

5 - HT
6 - E-HT
7 - HT
8 - E-HT

Input

9 - HT
10 - E-HT
11 - HT
12 - E-HT

Captured

13 - HT
14 - E-HT
15 - HT
16 - E-HT

Figure S4F - HaloTag

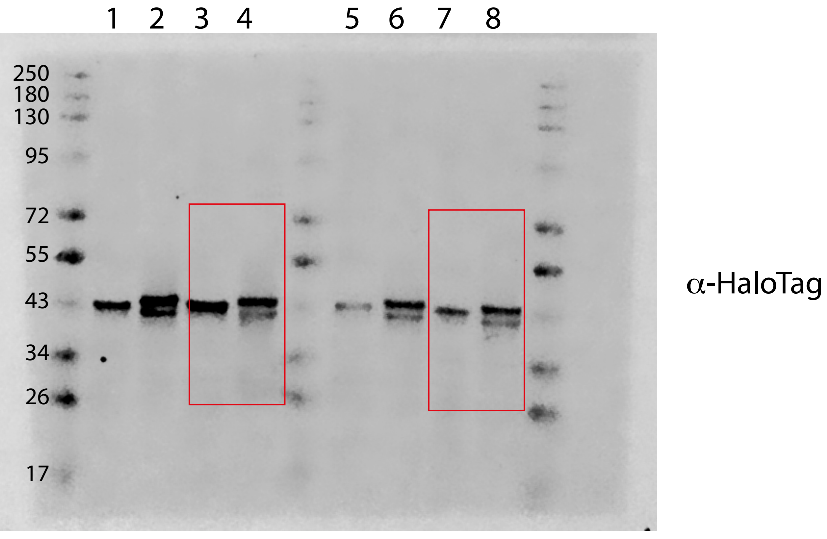

Western Blot Lanes

Input

1 - HT
2 - E-HT
3 - HT
4 - E-HT

Captured

5 - HT
6 - E-HT
7 - HT
8 - E-HT

Figure S4F - GRASP55

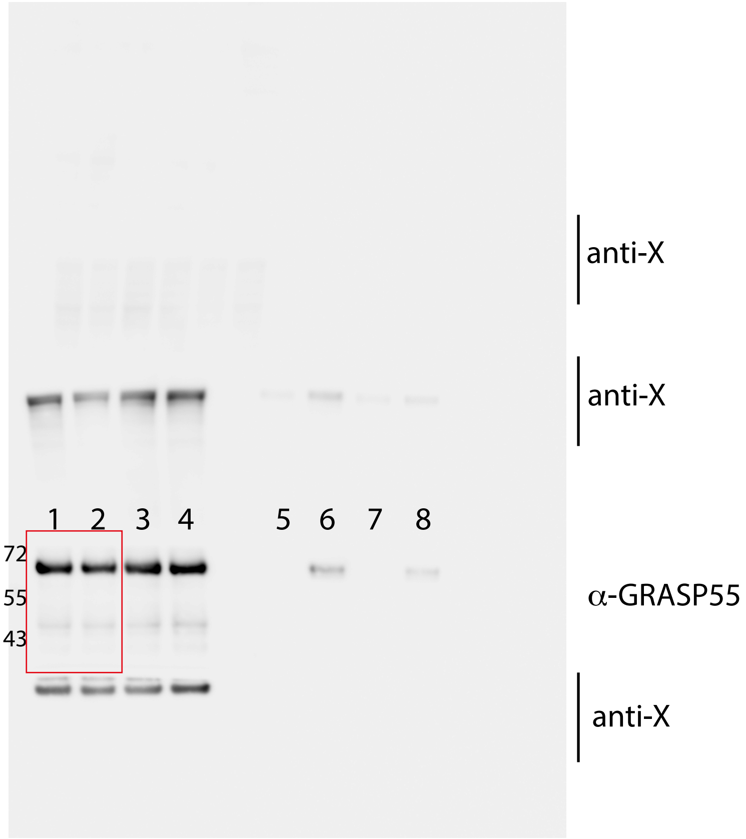

Low Exposure

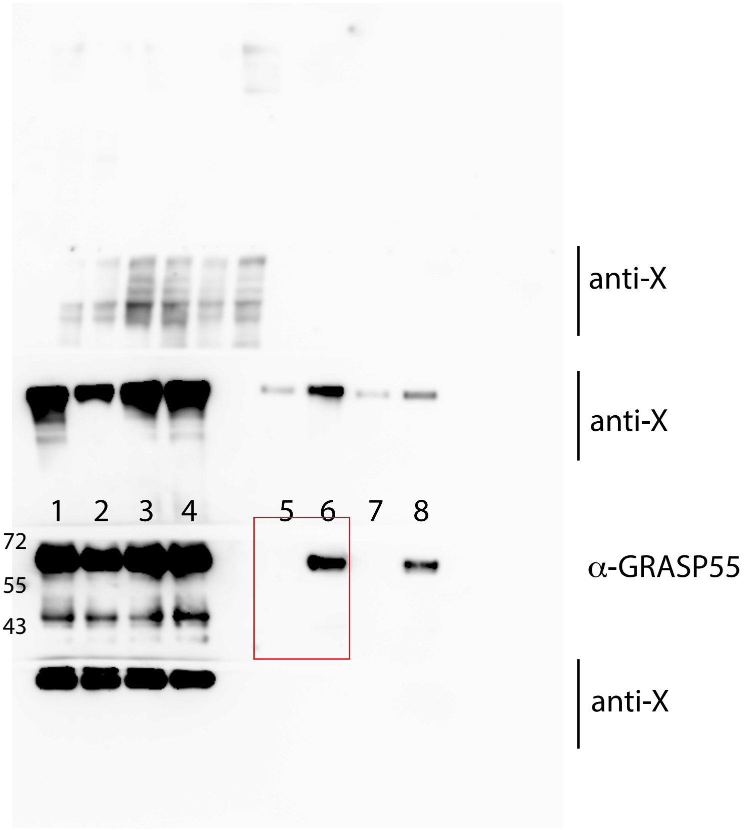

High Exposure

Western Blot Lanes

Input

1 - HT

2 - E-HT

3 - HT

4 - E-HT

Captured

5 - HT

6 - E-HT

7 - HT

8 - E-HT

Membranes were cut and exposed to different antibodies before reassembly for imaging.

GRASP55 blot marked up

Figure S9C

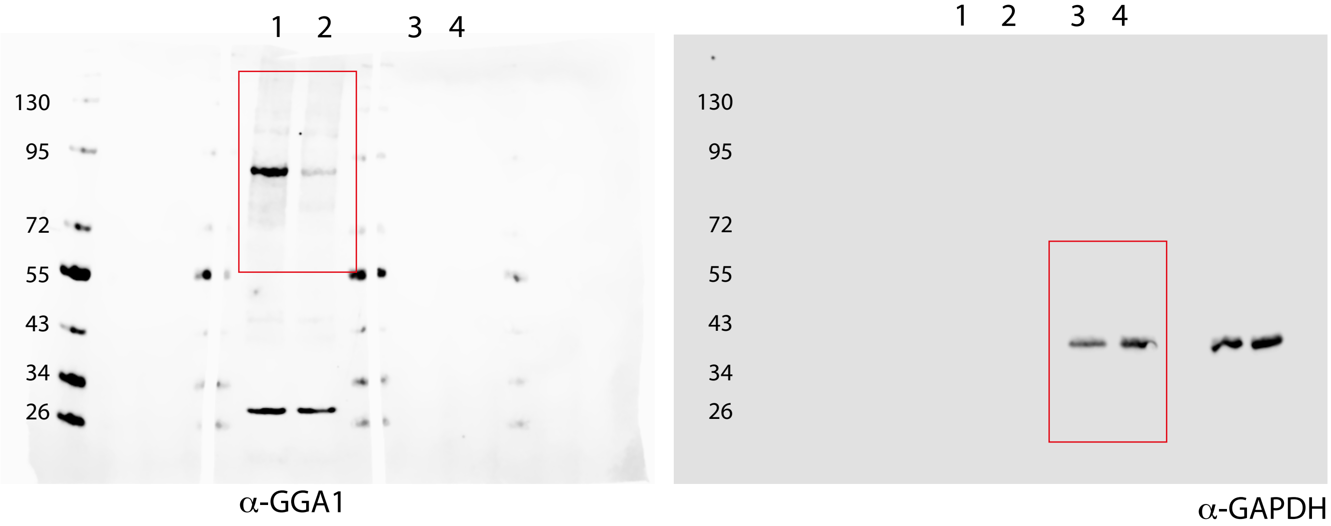

Western Blot Lanes
1 - VeroE6 lysate, control siRNA
2 - VeroE6 lysate, GGA1 siRNA
3 - VeroE6 lysate, control siRNA
4 - VeroE6 lysate, GGA1 siRNA

Figure S9C

High Exposure

Low Exposure

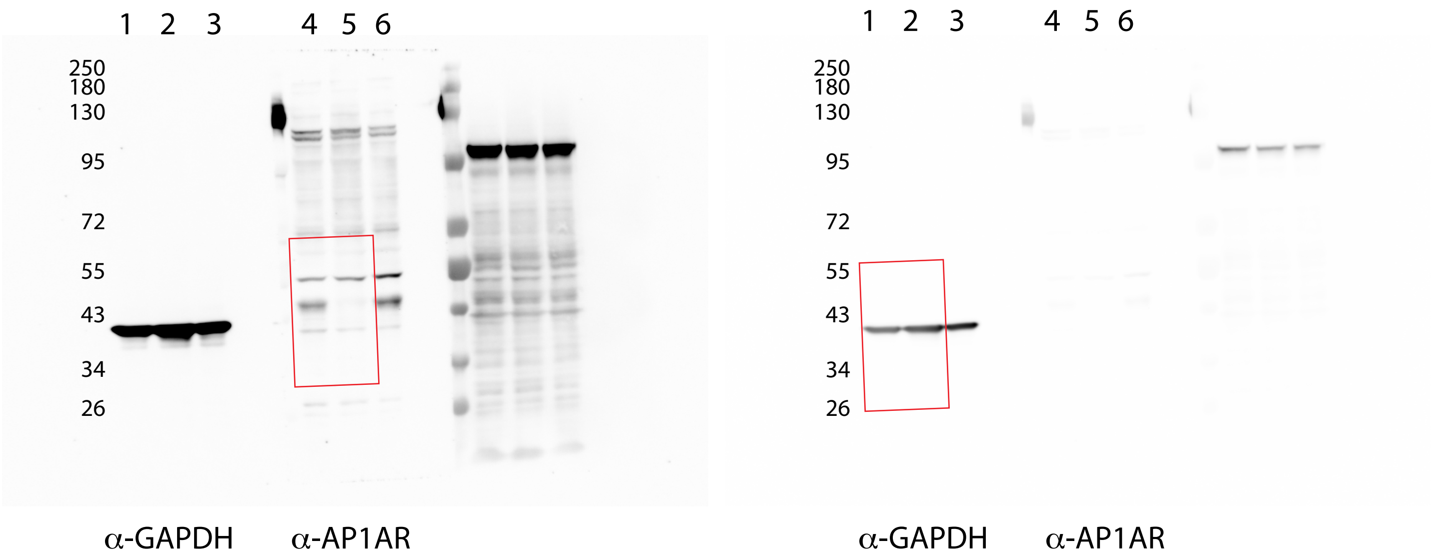

Western Blot Lanes
1 - VeroE6 lysate, control siRNA
2 - VeroE6 lysate, AP1AR siRNA
3 - VeroE6 lysate, AP1M1 siRNA
4 - VeroE6 lysate, control siRNA
5 - VeroE6 lysate, AP1AR siRNA
6 - VeroE6 lysate, AP1M1 siRNA

Figure S7B

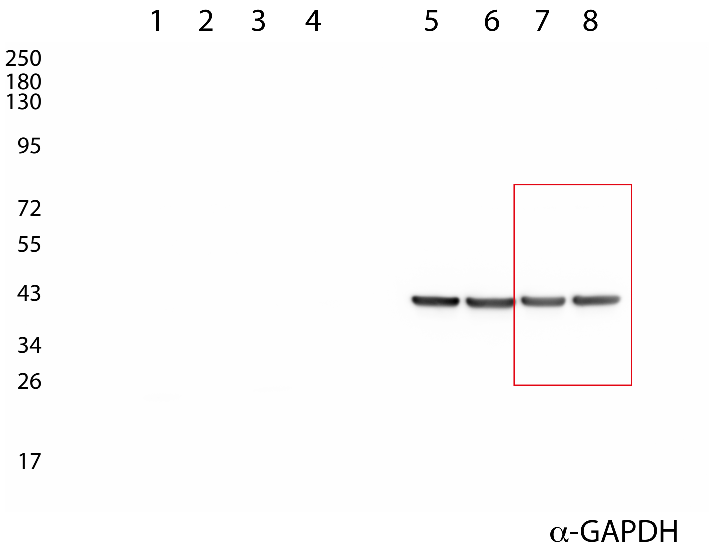

Western Blot Lanes
1 - Cal51 WT lysate
2 - Cal51 RER1 KO lysate
3 - VeroE6 WT lysate
4 - VeroE6 RER1 KO lysate
5 - Cal51 WT lysate
6 - Cal51 RER1 KO lysate
7 - VeroE6 WT lysate
8 - VeroE6 RER1 KO lysate

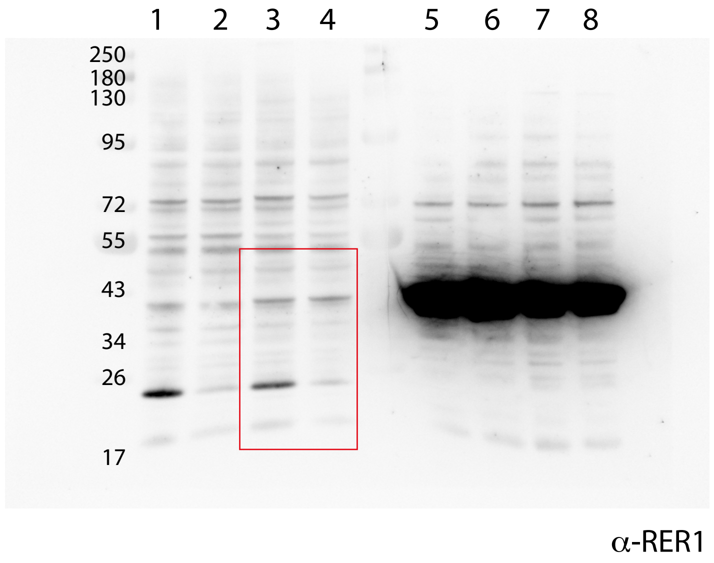

Figure S9C

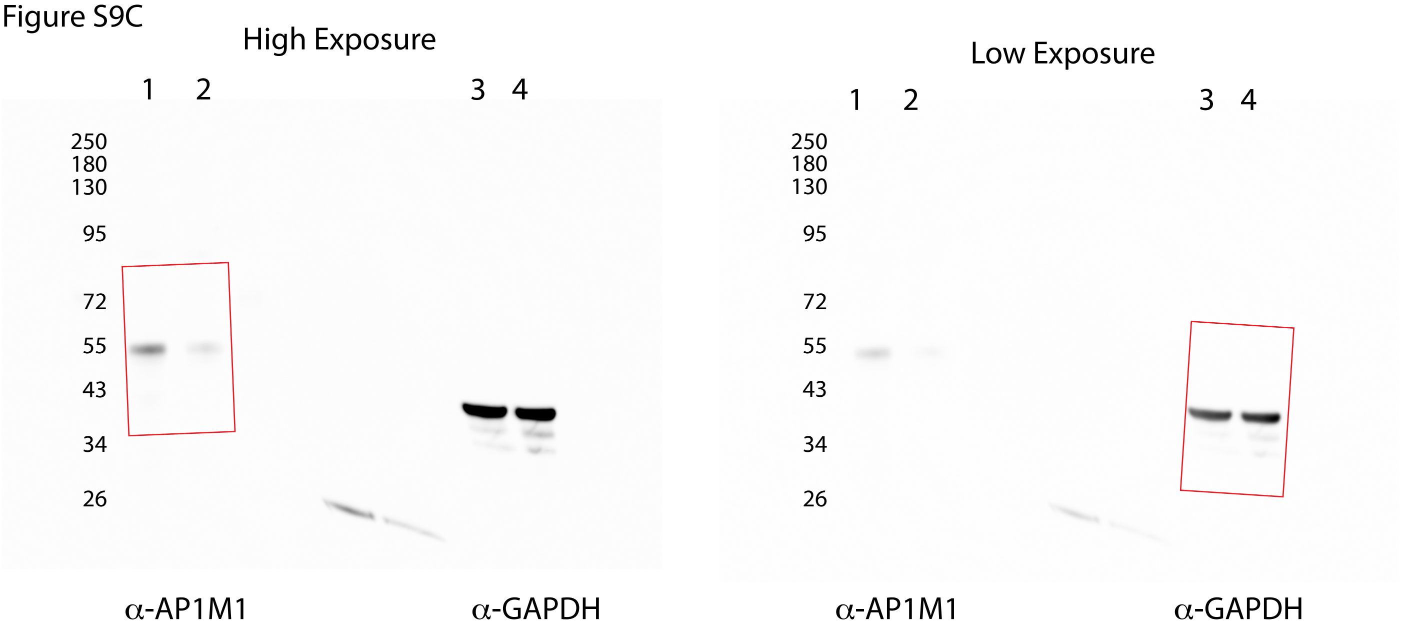

Western Blot Lanes
1 - VeroE6 lysate, control siRNA
2 - VeroE6 lysate, AP1M1 siRNA
3 - VeroE6 lysate, control siRNA
4 - VeroE6 lysate, AP1M1 siRNA

Figure S10D

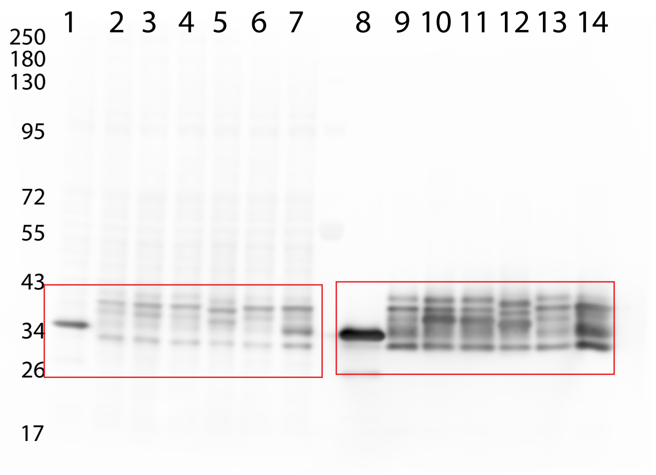

Anti-GFP:

Input	Captured
1 - GFP	8 - GFP
2 - E-Em WT	9 - E-Em WT
3 - E-Em M5	10 - E-Em M5
4 - E-Em FYK-AAA	11 - E-Em FYK-AAA
5 - E-Em Δ56-63	12 - E-Em Δ56-63
6 - E-Em Δ56-63:OC43	13 - E-Em Δ56-63:OC43
7 - E-Em Δ56-63:299E	14 - E-Em Δ56-63:299E

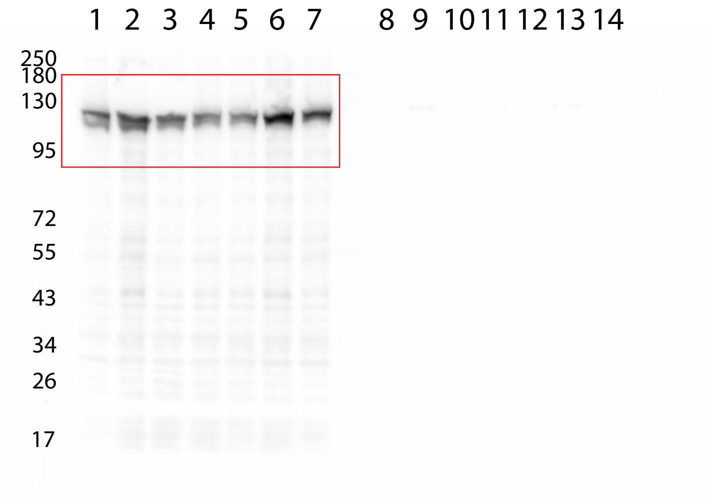

Anti-HA (low exposure):

Input	Captured
1 - GFP	8 - GFP
2 - E-Em WT	9 - E-Em WT
3 - E-Em M5	10 - E-Em M5
4 - E-Em FYK-AAA	11 - E-Em FYK-AAA
5 - E-Em Δ56-63	12 - E-Em Δ56-63
6 - E-Em Δ56-63:OC43	13 - E-Em Δ56-63:OC43
7 - E-Em Δ56-63:299E	14 - E-Em Δ56-63:299E

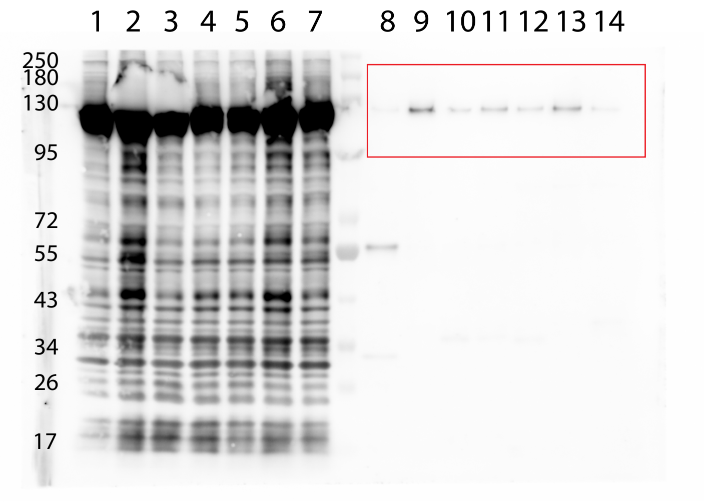

Anti-HA (high exposure):

Input	Captured
1 - GFP	8 - GFP
2 - E-Em WT	9 - E-Em WT
3 - E-Em M5	10 - E-Em M5
4 - E-Em FYK-AAA	11 - E-Em FYK-AAA
5 - E-Em Δ56-63	12 - E-Em Δ56-63
6 - E-Em Δ56-63:OC43	13 - E-Em Δ56-63:OC43
7 - E-Em Δ56-63:299E	14 - E-Em Δ56-63:299E

Figure S10A

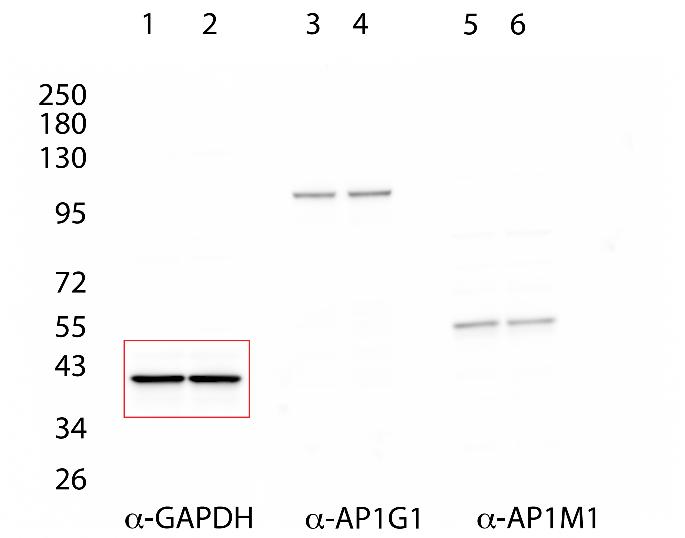

Low Exposure

Western Blot Lanes:
1 - VeroE6 WT lysate
2 - VeroE6 ARFRP1 KO lysate
3 - VeroE6 WT lysate
4 - VeroE6 ARFRP1 KO lysate
5 - VeroE6 WT lysate
6 - VeroE6 ARFRP1 KO lysate

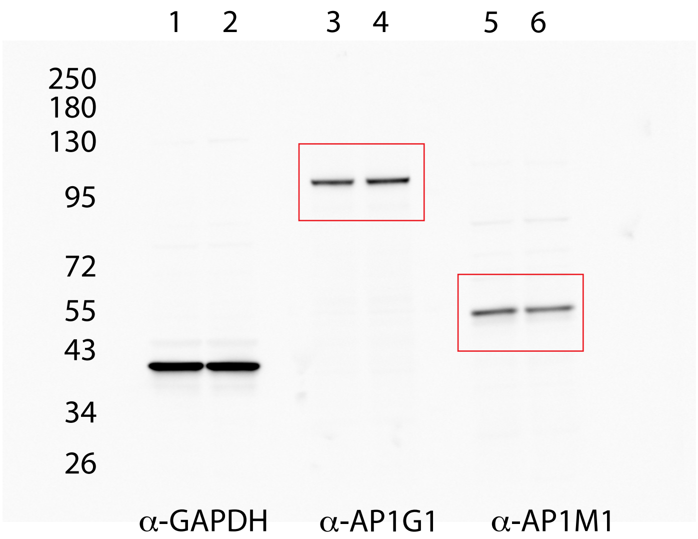

High Exposure
